# Flower colour and size-signals vary with altitude and resulting climate on the tropical-subtropical islands of Taiwan

**DOI:** 10.3389/fpls.2024.1304849

**Published:** 2024-02-01

**Authors:** Mani Shrestha, King-Chun Tai, Adrian G. Dyer, Jair E. Garcia, En-Cheng Yang, Anke Jentsch, Chun-Neng Wang

**Affiliations:** ^1^ Department of Disturbance Ecology and Vegetation Dynamics, Bayreuth Center of Ecology and Environmental Research (BayCEER), University of Bayreuth, Bayreuth, Germany; ^2^ Department of Life Science, National Taiwan University, Taipei, Taiwan; ^3^ Institute of Ecology and Evolutionary Biology, National Taiwan University, Taipei, Taiwan; ^4^ Department of Physiology, Monash University, Clayton, VIC, Australia; ^5^ Melbourne Data Analytics Platform, The University of Melbourne, Melbourne, VIC, Australia; ^6^ Department of Entomology, National Taiwan University, Taipei, Taiwan

**Keywords:** flora, flower colour, islands, green contrast, colour contrast, insect, vision

## Abstract

The diversity of flower colours in nature provides quantifiable evidence for how visitations by colour sensing insect pollinators can drive the evolution of angiosperm visual signalling. Recent research shows that both biotic and abiotic factors may influence flower signalling, and that harsher climate conditions may also promote salient signalling to entice scarcer pollinators to visit. In parallel, a more sophisticated appreciation of the visual task foragers face reveals that bees have a complex visual system that uses achromatic vision when moving fast, whilst colour vision requires slower, more careful inspection of targets. Spectra of 714 native flowering species across Taiwan from sea level to mountainous regions 3,300 m above sea level (a.s.l.) were measured. We modelled how the visual system of key bee pollinators process signals, including flower size. By using phylogenetically informed analyses, we observed that at lower altitudes including foothills and submontane landscapes, there is a significant relationship between colour contrast and achromatic signals. Overall, the frequency of flowers with high colour contrast increases with altitude, whilst flower size decreases. The evidence that flower colour signaling becomes increasingly salient in higher altitude conditions supports that abiotic factors influence pollinator foraging in a way that directly influences how flowering plants need to advertise.

## Introduction

Flowering plants form an experimentally accessible way to investigate how signals may evolve due to biotic influences like the visual capabilities of important pollinators (Chittka and Menzel, 1992; Dyer et al., 2012; Dyer et al., 2021) that facilitate the efficient transfer of pollen and/or abiotic factors like environmental climate or solar radiation (Koski and Galloway, 2018; Peach et al., 2020). Recent research has considered for the Australian continent the potential contributions of biotic or abiotic factors on flower colour and found that whilst both factors do contribute, the pollination by animal vectors was the main influence (Dalrymple et al., 2020).

Bees of the world are prolific pollinators of flowering plants (Michener, 2007). Comparative research on a wide variety of bee species from around the world has established that colour sensing in all tested bees is phylogenetically conserved, suggesting all known species probably see colour in a similar way (Briscoe and Chittka, 2001). The honeybee in particular serves as an important model species for understanding biotic pollination since colour vision has been extensively studied for over 100 years (von Frisch, 1914; von Frisch, 1967; von Helversen, 1972; Galizia et al., 2012). Honeybees have colour vision based on UV-, blue-, and green-sensitive photoreceptors which enable colour perception via opponent neural processing in multiple layers of the bee brain (Peitsch et al., 1992; Dyer et al., 2011). As free flying insects, the visual system of bees needs to balance several factors to forage including often moving longer distances, moving quickly between dense foliage, avoiding collisions (Srinivasan, 2010), detecting potential food sources like flowers amongst foliage (Bukovac et al., 2016; Dorin et al., 2023), and then enabling recognition of profitable flowers that are of value to land on (Garcia et al., 2020). As colour vision involves multiple photoreceptors and neural processing, this sense is more costly in terms of processing time. Honeybees thus employ their vision in a dynamic way. Flying fast and detecting targets at a distance (small visual angle) is processed by the green-sensitive photoreceptor channel enabling efficient achromatic vision, whilst colour vision is used when a bee slows down to inspect a flower at a large visual angle and this vision involves all three photoreceptor channels. Experiments on free flying bumblebees shows that both chromatic and achromatic signals and flower size significantly affect the efficiency with which either plastic-model (Spaethe et al., 2001) or real (Dyer et al., 2007) flowers are found. How this complexity of bee pollinator vision may have affected flower evolution has only been explored recently (e.g., Garcia et al., 2021), showing that in Nepal there is a significant positive correlation between flower size and chromatic colour contrast in the subalpine region, but a negative correlation at the lower altitudes; whilst at high elevations in Norway, flower size was positively correlated with achromatic green contrast. These initial results are suggestive that abiotic factors like climatic conditions may affect biotic flower colour signalling in complex ways.

To further investigate how abiotic factors may influence biotic flower colour signalling, island biogeography and the distribution of flowering plant diversity across the elevational gradients is important to understand. Taiwan, encompassing our current study sites, is an island territory close to mainland China and evolved approximately 4 to 5 million years ago (Wu, 1978; Biq et al., 1985). Several high mountains over 3,000 m a.s.l. exist in Taiwan and have created topographically isolated habitats below and above the treeline and fast-changing climatic zones (from tropical low land forest to alpine vegetation) along the elevation changes. Mountains are high value sites for understanding how abiotic or biotic factors may influence flower colour signalling (Arnold et al., 2009). The multiple origins of Taiwan flora (Huang, 2011) and complex island habitats (Li et al., 2013) thus may have potentially generated specific floral colour diversity at different elevations. For example, at different elevations, available resources such as soil nutrients as well as energy flux potential (Jentsch and White, 2019) may cause plants to have different flower sizes, which could require different spectral signalling to optimally capture the attention of bee pollinators. However, currently very little is known about Taiwanese or even Asian flower colouration and signal evolution with respect to animal pollinator colour vision. One recent study in Taiwan (Tai et al., 2020a) showed that flower signals do show the same general patterns of conforming to pollination by bees as has been established in other countries (Chittka and Menzel, 1992; Dyer et al., 2012; Bischoff et al., 2013; Shrestha et al., 2014). In addition, honeybees and bumblebees are frequently observed across Taiwan (Sung et al., 2006; Kudo et al., 2024 preprint). In the current study, we thus employed the analysis techniques of understanding how bee colour vision optimally finds flowers depending upon chromatic and/or achromatic signals, and the respective size of flowers. This research was conducted within a phylogenetically informed statistical analyses framework to investigate if there may be evidence of abiotic factors influencing biotic colour signalling at several elevational ranges.

## Materials and methods

### Study area and sample collection

**Figure 1:**
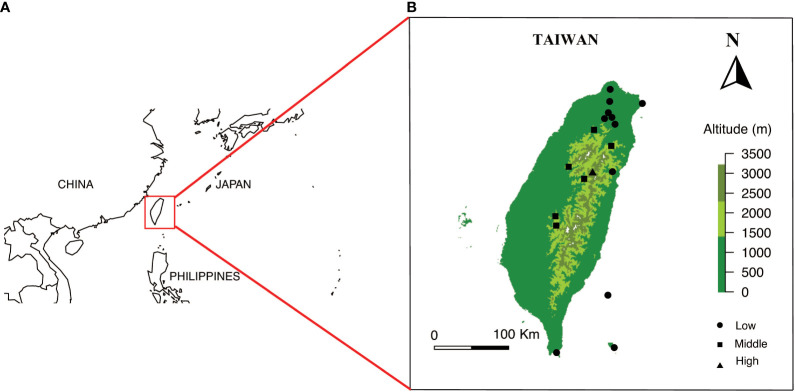
**(A)** Overview of location of Taiwan, and **(B)** Location of sampling sites and elevational range within Taiwan Solid circles, squares and triangle represents the different sampling sites. Maps are prepared using package Maps in R version 3.4.4.

**Figure 2:**
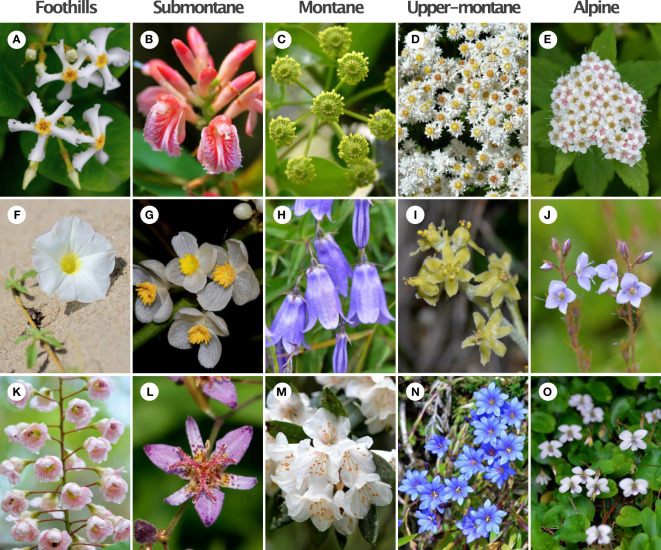
Example of flowering plants from five different elevational zones in Taiwan. **(A)** *Trachelospermum lanyuense* (15mm); **(B)** *Alpinia* x *ilanensis* (16mm); **(C)** *Trochodendron aralioides* (15mm); **(D)** *Anaphalis morrisonicola* (7mm); **(E)** *Spiraea hayatana* (4.5mm); **(F)** *Ipomoea imperati* (37.5mm); **(G)** *Begonia longifolia* (22mm); **(H)** *Adenophora morrisonensis* (31.5mm); **(I)** *Veratrum shuehshanarum* (12mm); **(J)** *Veronica morrisonicola* (11mm); **(K)** *Bretschneidera sinensis* (45mm); **(L)** *Tricyrtis formosana* (45mm); **(M)** *Rhododendron formosanum* (50mm); **(N)** *Gentiana arisanensis* (19.5mm); **(O)** *Viola adenothrix* var. *tsugitakaensis* (17.5mm). We show mean flower size (diameter, mm) in parenthesis for each species. Image credit: King-Chun Tai.

Our study sites include the islands of Taiwan. The main island of Taiwan lies in East Asia located in between 21.916107° N - 25.086991° N, 120.787456° E - 121.899446° E with a land area of 35,808 square kilometers. The island is surrounded by the South and East China Seas and at the nearest point is 130 km from any other major continental land mass (**Figure 1**, see details in Tai et al., 2020a). Our sample locations include the Taiwanese main island and two nearby offshore islands (Green Island and Orchid Island) (**Figure 1**). These sampling sites cover the major vegetation zones in Taiwan. Further, our study sites mostly includes the National Parks in the different elevational regions, which represents all vegetation types in the study region. We collected the flowers of native species (For example, **Figure 2**) from March 2016 to September 2017, covering two periods of the peak blooming (Tai, 2018). Our current study comprised of a total of 714 native flowering species.

Our study site was divided into five sub-categories based on vegetation zones (Li et al., 2013): i. Foothills (<500m a.s.l.), ii. Submontane (500-1,499m a.s.l.), iii. Montane (1,500-2,499m a.s.l.), iv. Upper-montane (2,500- 2,999m a.s.l.), and v. Alpine (>3,000m a.s.l.).

### Floral colour measurement

Floral colours were measured using an Ocean Optics spectrophotometer (Ocean Optics Inc., USB-4000+, USA) with a UV-VIS-NIR light source (Ocean Optics Inc., DH-2000-BAL, USA) and a quartz fiber-optic probe (Ocean Optics Inc., Lab-grade Reflection Probes, USA) relative to a 100% white standard and 0% black dark reference. The flower reflectance spectra were measured from 300 to 700 nm and then processed by the software OCEAN VIEW (Ocean Optics Inc., USA), and this range encompasses the visible spectrum of key pollinators. We sampled 3-5 flowers from each respective species from all of the sampling sites. All the flower colour data are available in Tai et al., 2020b (Dryad database).

### Hymenopteran colour space modelling

The hexagon colour space was developed for hymenopteran insects by Chittka (1992) and has recently been shown to accurately model broad-band flower spectra as perceived by bee pollinators (Dyer et al., 2007; Garcia et al., 2017). The implementation of the colour modelling used the photoreceptor data for *Apis melifera* since the spectral sensitivity of the photoreceptors and colour opponency mechanisms for higher order processing are well characterised (Peitsch et al., 1992; Dyer et al., 2011).

### Green contrast and colour contrast

Following the same method as Spaethe et al. (2001), the biologically relevant factors of the green contrast and/or colour contrast were modelled to represent the respective achromatic and chromatic signals of flowers for each species considering bee vision. Long wavelength green contrast modulated the green receptor excitation generated by any stimulus relative to the modelled 0.5 signal strength of the adaptation green foliage background in the hexagon colour space (Chittka, 1992; Spaethe et al., 2001; Garcia et al., 2021). Thus, the green contrast for each flower was calculated as an absolute difference value between the green receptor excitation and 0.5 (e.g., gc= |0.5-E(g)|), where E(g) represents the green receptor excitation. The colour contrast of each species’ flower was calculated as Euclidean distance from the respective locus to the achromatic centre in the bee colour hexagon (for calculation details, see Method S1).

### Phylogenetic tree

The phylogenetic tree constructed in Tai et al. (2020a), (available in the *Dryad Digital Repository*) was used to inform the analyses. We pruned the phylo-tree and prepared a new phylogenetic tree to exclude some species lacking flower size data. Some branches were left unresolved as polytomies.

**Figure 3:**
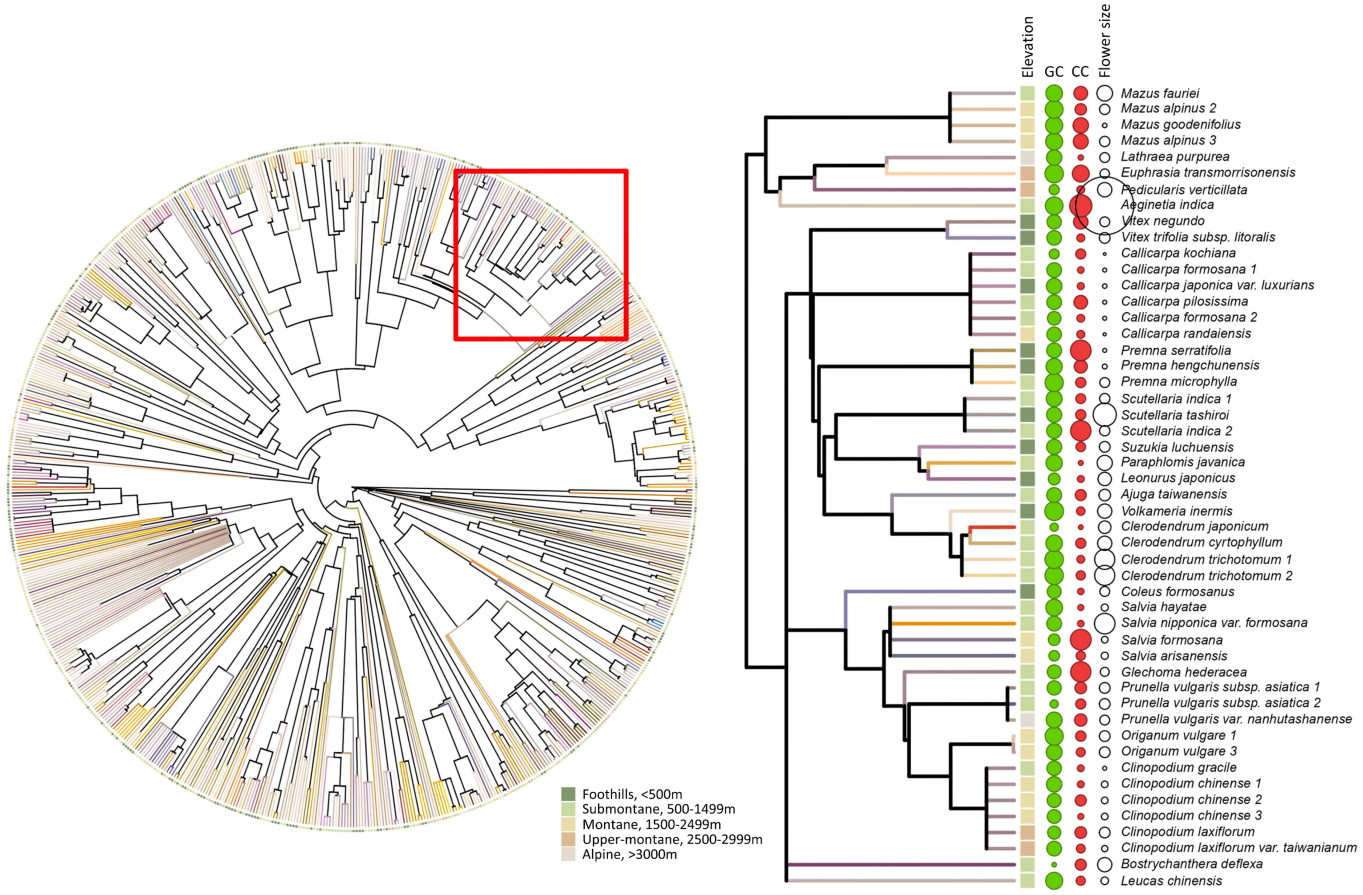
Left panel: phylogenetic relationship for 714 species. Terminal branches are visually presented as the flower colour of each species for human vision, which was generated based on the reflectance spectrum using the function ‘spec2rgb’ in R package PAVO. Solid circles at the tip represent the elevational zone for the respective plant species (dark green: foothills, light green: submontane, gold: montane, orange: upper-montane, beige: alpine). Right panel: pruned sub-tree from the whole phylogeny (see red square on the left panel) that shows the elevational zone, flower colour, and flower size for each example plant species. Solid squares at the tip represent the elevational zone. The solid green, solid red, and open white circles represent the green contrast, colour contrast, and flower size for each species, respectively, while the size of the circles indicates the magnitude of each variable.

To access the phylogenetic comparative analysis for flower signals data among five elevational zones, we used the phylo.maker function in R package ‘V.PhyloMaker’ (Jin and Qian, 2019). We used options ‘scenario 3’ to reconstruct the phylogeny and the output tree available is **Figure 3**.

### *Response variables:* flower size and elevation

Flower size (i.e., flower diameter, mm) and altitude data were obtained from various floras, books, and electronic databases (**Supplementary Table S2**). We basically used three electronic databases, including a) Flora of Taiwan, 2021 Second Edition (https://tai2.ntu.edu.tw), b) Flora of China, 2021 (www.efloras.org), and c) kplant, 2021 (http://kplant.biodiv.tw), to obtain the required data. Our data are based on Flora of Taiwan as we used local flora for the data extraction and used efloras or kplant when the required data was absent. The study obtained the minimum and maximum value of the flower diameter. If flower diameter data were absent, we alternatively obtained the minimum and maximum size of the most showy floral part, i.e., in most of the species, the petals, while in some clades, sepals (e.g., *Clematis* sp.) or standards (e.g., family Fabaceae). Flower size (Size) was thus typically measured as the average flower diameter, or 2 × average petal (or sepal) size of each species. Details of Size calculation for some atypical flowers are supplied in **Supplementary Table S2**.

**Figure 4:**
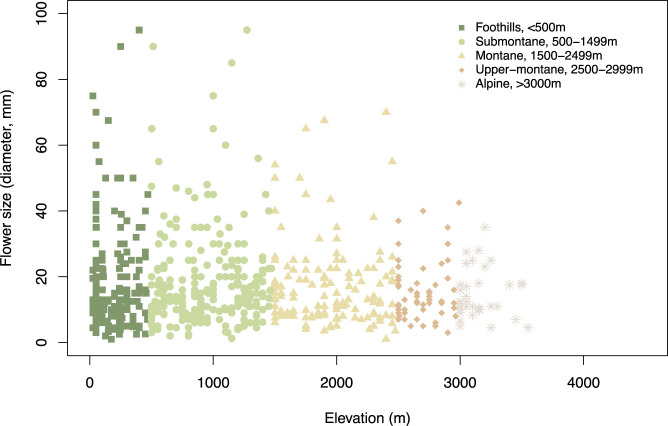
Flower size (i.e., flower diameter, mm) at different elevational zones.

We obtained the species distribution range for respective species using a similar approach and calculated the mean value (**Supplementary Table S2**) which is used in our data analysis. We categorised the respective species into five different groups based on the range of distribution for each species (see details above in *Study area and sample collection*). **Figure 4** provides the flower size across the different elevation zone.

### Data analysis

**Figure 5:**
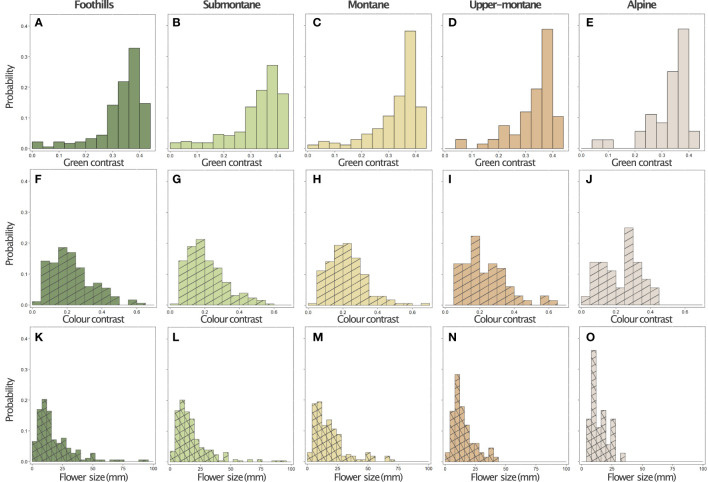
Distribution of green contrast (panels **A-E**, solid bars), colour contrast (panels **F-J**, hatched bars), and size (panels **K-O**, cross hatched bars) values for the flowers of 714 species at five elevational zones, n= in the island of Taiwan: Foothills (< 500 m a.s.l., first column, n=183), Submontane (500– 1,499 m a.s.l., second column, n=258), Montane (1,500–2,499 m a.s.l., third column, n=170), Upper-montane (2,500–2,999 m a.s.l., fourth column, n=67), and Alpine region (>3,000 m a.s.l., last column, n=36).

**Figure 6:**
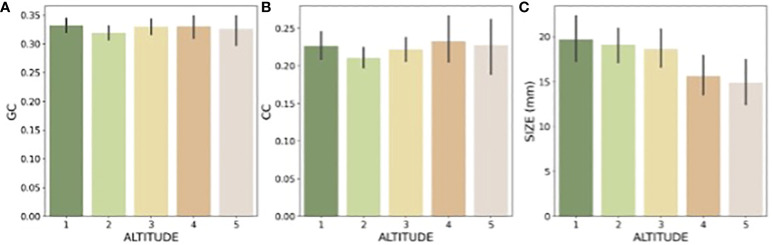
Mean and 95% confidence intervals for the (**A**: green contrast, GC), (**B**, colour contrast, CC), and (**C**: size) observed at each elevation: Foothills (1), Submontane (2), Montane (3), Upper-montane (4), and Alpine (5).

The graphical summary of the distribution of green contrast, colour contrast, and size values of flowers present at each location shows the variability of signals (**Figure 5**), and the quantitative data suggest that at the alpine altitude flowers display colour signals of the highest colour contrast, whilst the frequency of large flowers decreases (**Table 1**). This trend for flower size is reflected by the mean values observed for these parameters at each location (**Figure 6**), whilst colour contrast indicates that more complex processes perhaps influenced by different pollinators may be at play, and green contrast appears to be the least influenced by changes in altitude. The data showing that flower colour signals become increasingly salient at alpine altitudes is perhaps as a solution to facilitate the detection of smaller flower targets by bee pollinators. To test this hypothesis, we built linear models explaining the effect of either colour contrast or size on the amount of green contrast displayed by flowers sampled at each location. For each site, we defined green contrast values as the response variable and tested for any significant effect of either colour contrast or size on the dependent variable.

**Table 1:** Modes indicating to the most frequently observed green contrast (GC), colour contrast (CC), and size values at each elevational zone.

Location	GC	CC	Size (mm)
Foothills	0.373	0.168	14.4
Submontane	0.372	0.167	13.0
Montane	0.373	0.214	11.8
Upper-montane	0.368	0.161	11.4
Alpine	0.359	0.277	10.9

Following the analysis procedure in Garcia et al. (2021), we tested for the potential effects of colour contrast and size on the green contrast (to account for the pollinator discrimination perspective) of the data corresponding to each tested elevation using linear models. Each model had the general form *Y*=β_0_+β_1_ *X* +*E*, where Y represents the response variable (green contrast), X is a matrix containing the independent variable(s), colour contrast or size, included in the model, β_0_ is the intercept and β_1_ is the coefficient unique to each predictor, and *E* is an error term. The formulation of the linear models included reconstructed phylogenies for the species present on each site to account for non-independence between observations due to a shared evolutionary history (Freckleton et al., 2002). We calculated the regression coefficients for each elevation using the pgls function in R package ‘caper’ (Orme et al., 2018) which estimates the phylogenetic signal Pagel’s λ (Pagel, 1999) for each relation along with the value for the model’s parameters.

Pagel’s λ is a scaling parameter, ranging between 1 and 0, and was used to evaluate the degree to which closely related species exhibit similar trait values. When λ = 0, the trait is assumed to evolve independently along phylogeny (no signal) while λ=1 indicates the trait follows a Brownian motion evolution (strong signal). Some of our species tip label phylogeney are not resolved and have polytomies; Pagel’s λ performs more robustly with the incompletely resolved phylogeny in multispecies analysis (Molina-Venegas and Rodríguez, 2017). Thus, Pagel’s λ values were calculated for flower size and green and colour contrast. Modelling was done using the function phylosig in the package ‘phytools’ for R.

The analyses also tested for potential non-linear relationships between the independent variables and green contrast for each site. Potential non-linearity of the relationship between responses and the predictor variable was modelled by including the quadratic terms *Y* = β_0_+ β_1_ *X* ^2^ + β_2_ *X* + *E* into the models or by applying a logarithmic transformation to the independent variable *Y* = β_0_ + β_1_ *log*(*X*) + *E*. The model that best described the observations was selected based on the values of the Aikaike Information Criteria (AIC) and adjusted R^2^ value, following standard model selection procedures (Hurvich and Tsai, 1989; Crawley, 2013).

## Results

**Figure 7:**
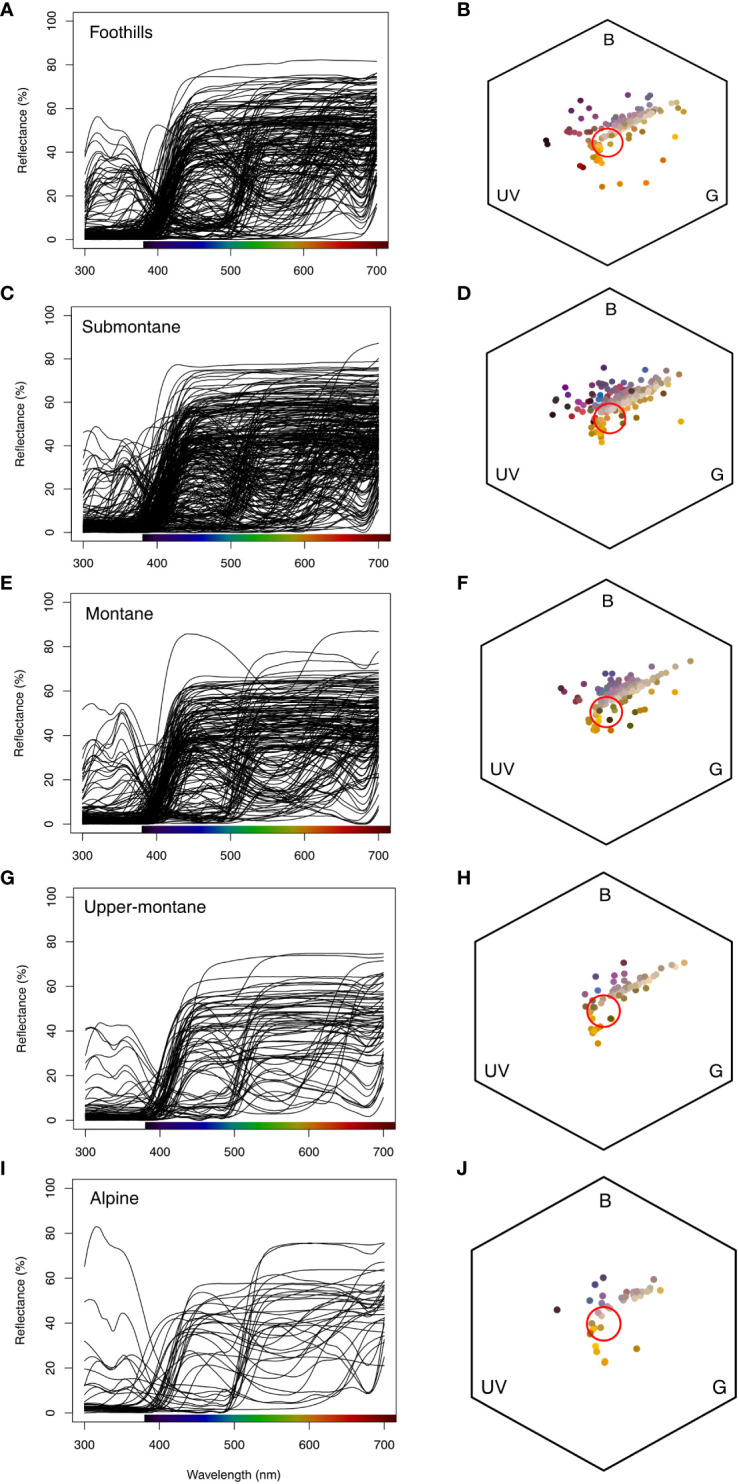
Floral reflectance spectra based on elevational zones (first column; **A, C, E, G, I**), and floral spectra in bee colour hexagon model in Taiwan (second column; **B, D, F, H, J**). The colour bar at the x-axis in the first column shows the human colour. The red circles in the hexagons show the achromatic region where bees do not reliably perceive chromatic signals. Colour solid circles (second column **B, D, F, H, J**) in bee hexagon represents the human flower colours that falls at different colour region (UV:Ultraviolet, B: and G: Green) of bee colour space.

We found that flower size decreases along the elevation zone (**Figure 4**) in our study sites. We further plotted the raw spectral data and converted them into the bee hexagon model (**Figure 7**) to show the how bees see the colour.

**Figure 8:**
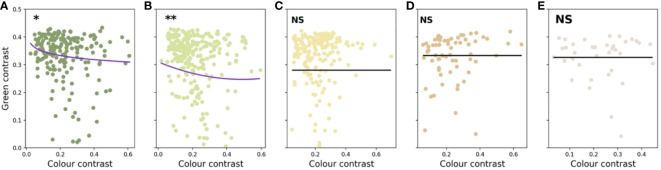
Graphical representation of the linear models describing the relationships between colour contrast, flower size, and green contrast for flowers present at five different elevations on the island of Taiwan: Foothills **(A)**, Submontane **(B)**, Montane **(C)**, Upper-montane **(D)**, and Alpine **(E)**. Significance of the relationship is indicated by asterisks: α = 0.05 (*), α = 0.01 (**), and not significant (NS). Markers on each panel indicate the values for the significant predictor and green contrast for the plant species present at each elevational zones.

The values and significance of the predictors in the five linear models are presented in **Table 2** along with the phylogenetic signal calculated for each data set. Colour contrast was found to have a significant non-linear effect on the green contrast of flowers present in the Foothills (< 500 m a.s.l.) and Submontane (500–1,499 m a.s.l.) zones (**Figure 8**). Flower size did not predict green contrast at any elevational zones.

**Table 2:** Values and statistical significance of the linear coefficients (β) describing the effect of colour contrast (cc) and flower size (sz) on the green contrast of flowers present at five different zones on the island of Taiwan.

Elevational zones	Parameters	Phylogenetic signal λ (95% CI)
Foothills	β Log(cc) = -0.021 (0.010); t = -2.04; P = 0.043* β sz = 6.21 x 10-4 (3.39 x 10 -4); t = 1.83, P = 0.068	0.274 (0.103, 0.502)
Submontane	βcc^2 =^ 0.260 (0.094); t = 2.76; P = 0.006** βcc = - 0.254 (0.093); t = -2.73; P = 0.007** βsz = 1.61x10-4 (3.77x10-4); t = -0.426; P = 0.670	0.182 (0.048, 0.380)
Montane	βcc = - 2.90x10-2 (5.52x10-2); t = -0.525; P = 0.601 βsz = 5.95x10-6- (5.60 x 10-4); t = 0.011; P = 0.992	0.838 (0.676, 0.925)
Upper-montane	βcc = 0.060 (0.074); t = 0.805; P = 0.424 βsz = -0.002 (0.001); t = -1.71; P = 0.091	0.239 (0.00, 0.629)
Alpine	βcc = -0.036 (0.127); t = 0.287; P = 0.776 βsz = 8.61x10-5 (1.91x10-3); t = 0.045; P = 0.964	0 (0.00, 0.996)

In Submontane zone, βcc2 is β2 and βcc is β1; there are two coefficients for the quadratic model; βcc is not the coefficient for the linear model here.

The values in parentheses following the coefficients indicate its standard error, and the asterisk indicates its significance at α = 0.05 (*) or α = 0.01 (**). The third column contains the phylogenetic signal for each model and its associated 95% confidence intervals.

The analyses revealed that alpine flowers show no phylogenetic signal for green and colour contrast, nor for flower size (**Table 3**). These results indicate that alpine zone flower colour and size signals are labile and may experience stronger selection pressure to be flexible and adapt when compared to some other altitudes. A phylogenetic signal for colour contrast was only detected for the montane species, whereas phylogenetic signals for green contrast were detected at all elevations except the alpine zone (**Table 3**). Overall, these results suggest that colour signals may be more evolutionary labile than the achromatic signals.

**Table 3:** Phylogenetic signals (Pagel’s λ) for the green contrast (GC), colour contrast (CC), and flower size (SIZE) of species among all five elevational zones in Taiwan.

Elevational zone	GC	CC	SIZE
Foothills	λ=0.288, p_(λ=0) _=0***	λ=0.217, p_(λ=0)_ =0.232	λ=0.170, p_(λ=0) _=0.164
Submontane	λ=0.168, p_(λ=0) _=0.013*	λ=0.214, p_(λ=0)_ =0.147	λ=0.594, p_(λ=0)_ =0***
Montane	λ=0.754, p_(λ=0) _=0***	λ=0.431, p_(λ=0)_ =0.005**	λ=0.956, p_(λ=0)_ =0***
Upper-montane	λ=0.231, p_(λ=0) _=0.045*	λ=0.166, p_(λ=0) _=0.267	λ=1.012, p_(λ=0) _=0***
Alpine	λ=0, p_(λ=0) _=1	λ=0, p_(λ=0) _=1	λ=0.508, p_(λ=0)_ =0.123

We also tested whether each Pagel’s λ is significantly deviated from 0 (no phylogenetic signal) based on the likelihood ratio, and the asterisks indicate the significance level for each test: *p<0.05, **p<0.01, ***p<0.001.

## Discussion

Recent research has demonstrated that both abiotic and biotic factors contribute to influencing the colouration of flower signals and suggested that more salient colours may be the product of harsher conditions (Dalrymple et al., 2020; Dyer et al., 2021). In parallel to this finding, evidence that important pollinators like bees use their vision in complex dynamic ways incorporating both achromatic (principally for flower detection at a distance) and chromatic (for flower recognition at close range) contrasts in a way that significantly affects flower colouration has emerged (Garcia et al., 2021). In a phylogenetically informed framework, we investigated if flower colour may change for a large island community depending upon the altitude of the flowering plants. This required both a large database of flowers and an accurate model of how colour signals are perceived by bee pollinators. Based on 714 flower spectra measured with a spectrophotometer from 300-700 nm and subsequent modelling in a hexagon colour space for plant-pollinator interactions (Chittka, 1992; Shrestha et al., 2019a), we found evidence of significant non-linear relationship between chromatic colour contrast and achromatic green contrast of flowers present in the foothills (< 500 m a.s.l.) and submontane (500–1,499 m a.s.l.) zones (**Figure 8**). Somewhat surprising given that achromatic green contrast enables the detection of flowers at a smaller visual angle due to the fundamental wiring of the visual system of honeybees (Giurfa et al., 1996; Giurfa et al., 1997; Spaethe et al., 2001; Dyer et al., 2008), our data analyses showed that flower size did not predict green contrast at any categorised elevation in Taiwan (**Table 3**). However, we did observe that the frequency of flowers with high colour contrast was highest at the alpine altitude, whilst flower size decreased at higher elevations (**Figure 4**). This finding for Taiwan is consistent with evidence from Australia (Dalrymple et al., 2020) and Norway (Garcia et al., 2021) that harsher environments such as on mountains influence flowering plants to advertise with more salient colour signals to best advertise to pollinators. This observation was additionally supported by the fact that the alpine flowers in Taiwan show no phylogenetic signals for neither green and colour contrast nor size (**Table 3**), which suggested elevation effects may drive selection not only of colour signals but also extend to the size signal.

Interestingly, it is worthy to note the colour contrast of the flowers show less phylogenetic signals compared to green contrast (**Table 3**), indicating colour contrast is more labile (**Figure 3**) and evolutionarily plastic. A similar pattern was observed within Australia as the phylogenetic signal was absent for colour contrast but detected when considering green contrast (Garcia et al., 2021). This is perhaps because the bees are known to employ green contrast for long distance detection but use colour contrast for close inspection for the flowers to visit or even pollinate, which has real impact on the reproduction success of angiosperms (Giurfa et al., 1996; Garcia et al., 2021). Therefore, colour contrast promoting a decision to land on a flower may theoretically receive stronger selection pressure than green contrast, although green contrast improves flower detection. It will thus be of value in future research to consider in other environments the degree to which the dynamics of bee pollinator vision may have influenced flower colour evolution in different countries.

The evidence that flower size overall decreased considering increasing altitudes (**Figure 4**) is interesting as behavioural studies over the past decade have shown that bees can use size as an important cue for making decisions (Avarguès-Weber et al., 2014; Howard et al., 2017), and interestingly, the capacity to process size is linked to the cognitive abilities of bees to estimate quantities (Bortot et al., 2020) and potentially forage efficiently. Moreover, similar to the patterns observed in Taiwan, flower size decreasing along elevation was reported at both species and community levels in China, Nepal, and Norway (Zhao and Wang, 2015; Garcia et al., 2021), which may be because of the resource-cost compromise in harsh alpine environments. Kudo et al. (2024) suggested that the higher frequency of flies compared to bees that visited flowering plants in the Mt. Hehuanshan area (Nantou County), 3000m a.s.l., in central Taiwan may be due to the harsh environmental conditions. However, in New Zealand, mountains bees were the main pollinator despite flies being more frequent flower visitors (Bischoff et al., 2013). Further, flower size reduction (**Figure 4**) may be due to the increasing self-pollination and the abrupt changes in climate at high elevations (Bliss, 1962; Körner, 2003; García – Camacho and Totland, 2009; Körner and Paulsen, 2009). Currently however, there is dearth of a long-term study about the later theory increasing selfing (Wirth et al., 2010) and potential reductions in flower sizes. We also found lower species diversity at higher elevations and our sampling size n =36 represents the overall species diversity at higher elevations. Nonetheless, several studies observed counter cases where some plant species present larger flower sizes at high altitudes (Kudo and Molau, 1999; He et al., 2017). Recent works in the northern and southern hemispheres provide comparable evidence that the level of precipitation contributes more than other factors to the variation of flower size (Zhao and Wang, 2015; Hendriks, 2019). Thus, complex changing environmental factors and the requirement to gather optimal rewards is likely to have been a driver for the complex visual processing of spatial cues like size that has been observed in bees (Avarguès-Weber et al., 2014; Howard et al., 2017; Bortot et al., 2020).

It is also known that flower signaling can be influenced by different orders or even species of pollinator. For example, whilst bee pollinators promote a variety of salient flower colours across the colour space of bees (**Figure 7**), plants pollinated by flies are most frequently a dull yellowish green colour and have loci in a different region of colour space compared to bee pollinated flowers (Garcia et al., 2022; Dyer et al., 2023). Interestingly, recent evidence suggests that in some mountain environments, fly visitors may become relatively more frequent than bee pollinators due to climatic conditions (McCabe and Cobb, 2021; Kudo et al., 2024). Even between bee species colour preference experiments reveal some evidence of consistent preferences, such as for short wavelength rich “blue” colours, but also some differences between species that might influence the frequency with which particular plants are pollinated (Giurfa et al., 1995; Koethe et al., 2016). A limitation of the current study is that whilst measuring flowering plant colouration across Taiwan, it was not possible to simultaneously capture a detailed survey of the insect species at the respective altitudes as this type of data in itself requires different methodologies (Shrestha et al., 2019b and reference within). We did however observe that honeybees, bumblebees, and several other bee species were frequently present across Taiwan (Starr, 1992; Sung et al., 2006; Lu and Huang, 2023; and Kudo et al., 2024); thus, the colorimetric analyses implemented was relevant at all field sites. Whilst for fly pollinators, new analyses tools like for colour similarity judgements have emerged (Garcia et al., 2022), it currently remains an open question how fly pollinators may use chromatic and/or achromatic contrasts for flower detection and subsequent recognition. Given the new colorimetric evidence that flower signalling does change dependent upon altitude (**Figure 4**), it will in future research be of high value to try and dissect flower visitations by insect species at different altitudes by insect pollinators in Taiwan, and to separately evaluate the dynamics of their vision using standard methods (Giurfa et al., 1996; Giurfa et al., 1997; Dyer et al., 2008; Dyer et al., 2016) and how such biotic-mediated processes can influence flower colour signalling at a community level (Shrestha et al., 2019c; Garcia et al., 2021). It is also potentially important to incorporate hypotheses frameworks that consider the complex signalling that may evolve when different species are flower visitors in an environment (Rodríguez-Sambruno et al., 2023).

When considering a large but relatively isolated study site like the island environments of Taiwan or Australia, it is likely that abiotic factors will interact with biotically mediated colour selection forces in a complex and non-trivial way (Verloop et al., 2020). For example, variations in solar radiation and energy flux potential (Jentsch and White, 2019) can lead to a modulation of the production of anthocyanins and flavonols (Falcone Ferreyra et al., 2012), which could also influence flower appearance. Various pigments may also have specific functions in protecting flowers from abiotic stressors like increasing the amount of ultraviolet absorbing pigments to mask the amount of potentially damaging ultraviolet light that is reflected to the pollen (Koski and Ashman, 2015; Dyer et al., 2021). Pigments within the petal surface can affect the resultant colour signals in complex ways due to how and where within the petal surface the pigments are incorporated relative to other optical-modulating particles or cell structures (van der Kooi et al., 2019). The current study demonstrates that flower colour and its pattern does exhibit evidence of significant variations in signalling at different elevations across Taiwan as perceived by biologically important bee pollinators. Therefore, it will be of high value to conduct more fine-grained research within specific altitudinal ranges to understand the precise mechanisms that are involved, and how possible alterations in the climate and/or habitat fragmentation may impact them.

## Data availability statement

Dryad Digital Repository: [http://doi.org/10.5061/dryad.63xsj3v08, Tai et al., 2020b].

## Author contributions

MS: Conceptualization, Data curation, Formal analysis, Investigation, Methodology, Project administration, Resources, Software, Supervision, Validation, Visualization, Writing – original draft, Writing – review & editing, Funding acquisition. K-CT: Data curation, Formal analysis, Investigation, Methodology, Validation, Visualization, Writing – original draft, Writing – review & editing, Conceptualization, Software. AD: Conceptualization, Formal analysis, Funding acquisition, Investigation, Methodology, Supervision, Validation, Visualization, Writing – original draft, Writing – review & editing, Software. JG: Formal analysis, Investigation, Methodology, Validation, Visualization, Writing – original draft, Writing – review & editing. E-CY: Investigation, Resources, Supervision, Visualization, Writing – review & editing. AJ: Investigation, Resources, Supervision, Visualization, Writing – review & editing, Funding acquisition, Validation. C-NW: Conceptualization, Data curation, Funding acquisition, Investigation, Project administration, Resources, Supervision, Validation, Visualization, Writing – review & editing, Methodology.

## Supplementary material

Supplementary Table 1 Phylogenetic tree of 714 species, nexus file, named as “tw714tree pruned Tai 2020.nex”.

Supplementary Table 2 Table_S2_Size_gc_cc_data_species_list.csv.

Supplementary method S1 Calculation details for green contrast and colour contrast.
