# Supplementary material for: Flower colour and size-signals vary with altitude and resulting climate on the tropical-subtropical islands of Taiwan
Source: Front Plant Sci. 2024 Feb 1;15:1304849. doi: 10.3389/fpls.2024.1304849 (PMC10867191; doi:10.3389/fpls.2024.1304849)
Supplement: Supplementary Table 1 — Phylogenetic tree of 714 species, nexus file, named as “tw714tree pruned Tai 2020.nex”. [file Table_1.docx]

#NEXUS

[R-package PHYTOOLS, Thu Sep 28 10:50:31 2023]

BEGIN TAXA;

DIMENSIONS NTAX = 714;

TAXLABELS

Abelia_chinensis_var._ionandra

Abutilon_indicum_var._guineense

Acanthephippium_striatum

Acer_caudatifolium

Acer_rubescens

Aconitum_fukutomei

Acronychia_pedunculata

Actinidia_setosa

Adenophora_morrisonensis

Adenophora_morrisonensis_subsp._uehatae_1

Adenophora_morrisonensis_subsp._uehatae_3

Adenophora_triphylla

Aeginetia_indica

Aeschynanthus_acuminatus

Agrimonia_pilosa

Ainsliaea_latifolia_subsp._henryi

Ajuga_taiwanensis

Akebia_longiracemosa

Alangium_chinense

Aletris_spicata

Alniphyllum_pterospermum_1

Alniphyllum_pterospermum_2

Alpinia_flabellata

Alpinia_intermedia

Alpinia_japonica

Alpinia_koshunensis

Alpinia_kusshakuensis

Alpinia_pricei

Alpinia_sessiliflora

Alpinia_shimadae

Alpinia_x_ilanensis

Alpinia_zerumbet

Alysicarpus_vaginalis

Amitostigma_alpestre

Anaphalis_morrisonicola_2

Anaphalis_morrisonicola_3

Anaphalis_nepalensis

Androsace_umbellata

Anemone_vitifolia_2

Anemone_vitifolia_3

Anneslea_fragrans_var._lanceolata

Anodendron_benthamianum

Arabidopsis_lyrata_subsp._kamchatica

Ardisia_cornudentata

Ardisia_cornudentata_subsp._morrisonensis

Ardisia_cornudentata_var._stenosepala

Ardisia_elliptica

Ardisia_humilis

Ardisia_japonica

Ardisia_polysticta

Ardisia_quinquegona

Ardisia_sieboldii

Ardisia_squamulosa

Argostemma_solaniflorum

Arisaema_consanguineum

Arisaema_formosanum_2

Arisaema_heterophyllum

Arisaema_ringens

Aristolochia_cucurbitifolia

Aristolochia_shimadae_1

Aristolochia_shimadae_2

Aristolochia_zollingeriana

Arundina_graminifolia

Asarum_chatienshanianum

Asarum_hypogynum

Asarum_macranthum

Aster_ageratoides

Aster_indicus

Aster_oldhamii

Aster_taiwanensis

Astilbe_longicarpa

Astronia_ferruginea

Aucuba_japonica

Bacopa_monnieri

Balanophora_laxiflora

Barbarea_orthoceras

Barringtonia_racemosa

Barthea_barthei

Bauhinia_championii

Begonia_chitoensis

Begonia_fenicis

Begonia_formosana

Begonia_formosana_2

Begonia_laciniata_1

Begonia_laciniata_2

Begonia_longifolia

Berberis_brevisepala

Berberis_kawakamii

Berberis_mingetsensis

Berberis_morrisonensis

Blastus_cochinchinensis

Bletilla_formosana

Boenninghausenia_albiflora

Bostrychanthera_deflexa

Bredia_oldhamii

Bredia_scandens

Bretschneidera_sinensis

Caesalpinia_crista

Calanthe_aristulifera

Calanthe_puberula

Calanthe_sylvatica

Calanthe_triplicata

Callicarpa_formosana_1

Callicarpa_formosana_2

Callicarpa_japonica_var._luxurians

Callicarpa_kochiana

Callicarpa_pilosissima

Callicarpa_randaiensis

Calophyllum_blancoi

Calophyllum_inophyllum

Camellia_brevistyla

Camellia_euryoides_var._nokoensis

Camellia_furfuracea

Camellia_hengchunensis

Camellia_japonica

Camellia_tenuiflora

Canavalia_lineata

Canavalia_rosea

Cardiandra_formosana

Carpesium_nepalense_2

Carpesium_nepalense_3

Celastrus_punctatus

Centaurium_japonicum

Cephalanthera_alpicola

Cephalantheropsis_obcordata

Cerastium_morrisonense_2

Cerastium_morrisonense_3

Cerbera_manghas

Chamaecrista_garambiensis

Cheilotheca_humilis_2

Cheilotheca_humilis_3

Cheilotheca_macrocarpa

Cheirostylis_octodactyla

Chimaphila_japonica

Chionanthus_ramiflorus

Chionanthus_retusus

Cirsium_brevicaule

Cirsium_japonicum_var._australe

Cirsium_suzukii

Citrus_reticulata_var._depressa

Clematis_chinensis

Clematis_grata_1

Clematis_grata_2

Clematis_lasiandra

Clematis_montana

Clematis_tashiroi

Clematis_tsugetorum

Clerodendrum_cyrtophyllum

Clerodendrum_japonicum

Clerodendrum_trichotomum_1

Clerodendrum_trichotomum_2

Clinopodium_chinense_1

Clinopodium_chinense_2

Clinopodium_chinense_3

Clinopodium_gracile

Clinopodium_laxiflorum

Clinopodium_laxiflorum_var._taiwanianum

Codonacanthus_pauciflorus

Codonopsis_kawakamii

Coleus_formosanus

Commelina_auriculata

Commelina_communis

Commelina_diffusa

Conandron_ramondioides

Coptis_quinquefolia

Corydalis_ochotensis

Corydalis_ophiocarpa

Corydalis_pallida

Corydalis_tashiroi_1

Corydalis_tashiroi_2

Cotoneaster_konishii

Cotoneaster_morrisonensis

Cremastra_appendiculata_var._variabilis

Crepidiastrum_taiwanianum

Crepidium_matsudae

Crossostephium_chinense

Crotalaria_similis

Cymbidium_ensifolium

Cymbidium_sinense

Cynanchum_lanhsuense

Cynanchum_ovalifolium

Cynoglossum_alpestre

Cynoglossum_furcatum

Cypripedium_debile

Damnacanthus_indicus

Daphne_arisanensis_2

Daphne_arisanensis_3

Daphne_kiusiana_var._atrocaulis

Decaspermum_gracilentum

Dendrobium_catenatum

Dendrobium_goldschmidtianum

Dendrolobium_umbellatum

Derris_oblonga

Deutzia_pulchra_1

Deutzia_pulchra_2

Deutzia_pulchra_3

Deutzia_taiwanensis

Dianella_ensifolia

Dianthus_pygmaeus_2

Dianthus_pygmaeus_3

Dianthus_superbus_subsp._longicalycinus

Diospyros_discolor

Diospyros_maritima

Disporum_nantouense

Disporum_sessile

Donax_canniformis

Ehretia_philippinensis

Elaeagnus_formosensis

Elaeagnus_umbellata

Elaeocarpus_argenteus

Elaeocarpus_japonicus

Ellisiophyllum_pinnatum_2

Ellisiophyllum_pinnatum_3

Embelia_laeta_subsp._papilligera

Enkianthus_perulatus

Epilobium_amurense_2

Epilobium_amurense_3

Epilobium_brevifolium_subsp._trichoneurum

Epilobium_hohuanense

Erigeron_fukuyamae

Erigeron_morrisonensis

Eriobotrya_deflexa_f._koshunensis

Erythrina_variegata

Eulophia_pulchra

Eulophia_pulchra_var._actinomorpha

Euonymus_cochinchinensis

Euonymus_japonicus

Euphorbia_jolkinii

Euphrasia_transmorrisonensis

Eurya_chinensis

Evolvulus_alsinoides

Farfugium_japonicum

Filipendula_kiraishiensis

Fissistigma_glaucescens

Flacourtia_rukam

Fragaria_hayatai

Galactia_tashiroi

Galeola_falconeri

Garcinia_multiflora

Garcinia_subelliptica

Gardenia_jasminoides

Gaultheria_borneensis_2

Gaultheria_borneensis_3

Gaultheria_leucocarpa_var._cumingiana

Geniostoma_rupestre

Gentiana_arisanensis

Gentiana_davidii_var._formosana_2

Gentiana_davidii_var._formosana_3

Gentiana_flavomaculata_2

Gentiana_flavomaculata_3

Gentiana_flavomaculata_var._yuanyanghuensis

Gentiana_scabrida_var._punctulata

Geophila_repens

Geranium_hayatanum

Geranium_robertianum

Geranium_suzukii

Geranium_thunbergii

Geranium_wilfordii

Glechoma_hederacea

Glochidion_lanceolatum

Glochidion_rubrum

Glossocardia_bidens

Goniothalamus_amuyon

Goodyera_foliosa

Goodyera_nankoensis

Goodyera_procera

Goodyera_rubicunda

Gordonia_axillaris

Guettarda_speciosa

Gymnema_sylvestre

Gymnosporia_emarginata

Gynura_elliptica

Hedyotis_strigulosa_var._parvifolia

Heliotropium_indicum

Heloniopsis_umbellata_1

Heloniopsis_umbellata_2

Helwingia_japonica_subsp._formosana

Hemiboea_bicornuta_1

Hemiboea_bicornuta_2

Hemigraphis_reptans

Hemiphragma_heterophyllum

Heritiera_littoralis

Hernandia_nymphaeifolia

Hewittia_malabarica

Hibiscus_mutabilis

Hibiscus_taiwanensis_1

Hibiscus_taiwanensis_2

Hibiscus_tilliaceus

Hieracium_morii

Houttuynia_cordata_1

Houttuynia_cordata_2

Hybanthus_enneaspermus

Hydrangea_angustipetala

Hydrangea_aspera

Hydrangea_chinensis_1

Hydrangea_chinensis_2

Hydrangea_integrifolia

Hygrophila_pogonocalyx

Hylodesmum_leptopus

Hypericum_formosanum_1

Hypericum_formosanum_2

Hypericum_nagasawae

Hypericum_subalatum

Hypericum_taihezanense

Hypoestes_cumingiana

Hypoestes_purpurea

Ilex_asprella

Ilex_ficoidea

Ilex_kusanoi

Ilex_maximowicziana

Illicium_anisatum

Illicium_arborescens

Impatiens_tayemonii

Impatiens_uniflora_2

Impatiens_uniflora_3

Indigofera_byobiensis

Indigofera_pedicellata

Indigofera_ramulosissima

Indigofera_spicata

Indigofera_trifoliata

Indigofera_zollingeriana

Ipomoea_imperati

Ipomoea_littoralis

Ipomoea_pes-caprae_subsp._brasiliensis

Iris_domestica

Itea_parviflora

Ixeridium_laevigatum

Ixeris_chinensis

Jasminanthes_mucronata

Jasminum_nervosum

Justicia_procumbens_1

Justicia_procumbens_2

Justicia_procumbens_var._hayatae

Kalanchoe_garambiensis

Kalanchoe_integra

Kleinhovia_hospita

Koelreuteria_elegans_subsp._formosana

Lagerstroemia_subcostata

Lasianthus_attenuatus

Lasianthus_fordii

Lathraea_purpurea

Leea_guineensis

Leea_philippinensis

Leontopodium_microphyllum

Leonurus_japonicus

Lespedeza_formosa

Leucas_chinensis

Ligularia_kojimae

Ligustrum_liukiuense

Ligustrum_sinense

Lilium_longiflorum_var._formosanum_1

Lilium_longiflorum_var._formosanum_3

Lilium_longiflorum_var._scabrum

Limnophila_aromatica

Lindernia_ruellioides

Liparis_bootanensis

Liparis_formosana

Liparis_gigantea

Liparis_henryi

Liparis_sootenzanensis

Liriope_muscari

Litsea_cubeba

Lobelia_nummularia_1

Lobelia_nummularia_2

Lobelia_seguinii

Lonicera_acuminata_2

Lonicera_acuminata_3

Lonicera_apodantha

Lonicera_japonica_1

Lonicera_japonica_2

Ludwigia_octovalvis

Lycianthes_biflora

Lycianthes_lysimachioides

Lysimachia_ardisioides_1

Lysimachia_ardisioides_2

Lysimachia_congestiflora

Lysimachia_mauritiana

Lysimachia_remota

Lysionotus_pauciflorus_1

Lysionotus_pauciflorus_2

Maesa_japonica

Maesa_lanyuensis

Maianthemum_formosanum

Marsdenia_formosana

Mazus_alpinus_2

Mazus_alpinus_3

Mazus_fauriei

Mazus_goodenifolius

Medinilla_fengii_1

Medinilla_fengii_2

Medinilla_formosana

Melanolepis_multiglandulosa

Melastoma_malabathricum

Melicope_triphylla

Melodinus_angustifolius

Memecylon_lanceolatum

Merremia_gemella

Michelia_compressa_var._formosana

Michelia_compressa_var._lanyuensis

Millettia_pulchra_var._microphylla

Millettia_reticulata

Molineria_capitulata

Moneses_uniflora

Monotropa_hypopitys

Morinda_citrifolia

Mucuna_macrocarpa

Mucuna_membranacea

Murdannia_loriformis

Murraya_crenulata

Murraya_paniculata_var._omphalocarpa

Mussaenda_formosanum

Mussaenda_macrophylla

Mussaenda_parviflora

Mussaenda_taihokuensis_1

Mussaenda_taihokuensis_2

Myristica_ceylanica

Myristica_elliptica_var._simiarum

Myrmechis_drymoglossifolia

Neanotis_formosana

Nemosenecio_formosanus

Neottia_formosana

Neottia_meifongensis

Nothapodytes_nimmoniana

Odontochilus_bisaccatus

Olax_imbricata

Ophiopogon_intermedius_1

Ophiopogon_intermedius_2

Ophiopogon_intermedius_3

Ophiorrhiza_japonica

Ophiorrhiza_kuroiwae

Ophiorrhiza_pumila

Origanum_vulgare_1

Origanum_vulgare_3

Osteomeles_anthyllidifolia

Oxalis_acetosella_2

Oxalis_acetosella_3

Palaquium_formosanum

Paraphlomis_javanica

Paraprenanthes_sororia

Paris_polyphylla_var._stenophylla_2

Paris_polyphylla_var._stenophylla_3

Parnassia_palustris_2

Parnassia_palustris_3

Parsonsia_alboflavescens

Patrinia_glabrifolia

Pedicularis_verticillata

Pemphis_acidula

Pentacoelium_bontioides

Peracarpa_carnosa_2

Peracarpa_carnosa_3

Peristrophe_bivalvis

Peristrophe_japonica

Persicaria_chinensis

Petasites_formosanus_2

Petasites_formosanus_3

Phaius_mishmensis

Phaius_tankervilleae

Philydrum_lanuginosum

Photinia_beauverdiana

Photinia_lucida

Photinia_niitakayamensis

Picris_morrisonensis_2

Picris_morrisonensis_3

Pieris_japonica

Pittosporum_illicioides

Pittosporum_pentandrum

Platanthera_brevicalcarata

Platanthera_mandarinorum_subsp._pachyglossa_2

Platanthera_mandarinorum_subsp._pachyglossa_3

Platanthera_sachalinensis

Polygala_sibirica

Polygonatum_arisanense_1

Polygonatum_arisanense_2

Polygonum_biconvexum

Polygonum_cuspidatum_2

Polygonum_cuspidatum_3

Polygonum_posumbu

Polygonum_runcinatum

Pongamia_pinnata

Portulaca_oleracea

Portulaca_pilosa

Potentilla_leuconota

Potentilla_matsumurae_var._pilosa

Premna_hengchunensis

Premna_microphylla

Premna_serratifolia

Primula_miyabeana

Prinsepia_scandens

Prunella_vulgaris_subsp._asiatica_1

Prunella_vulgaris_subsp._asiatica_2

Prunella_vulgaris_var._nanhutashanense

Prunus_campanulata

Prunus_phaeosticta

Prunus_salicina

Prunus_transarisanensis

Pueraria_montana_1

Pueraria_montana_2

Pyracantha_koidzumii_1

Pyracantha_koidzumii_2

Pyrola_morrisonensis

Radermachera_sinica

Ranunculus_cantoniensis_1

Ranunculus_cantoniensis_2

Ranunculus_formosa-montanus

Ranunculus_junipericola

Ranunculus_sieboldii

Ranunculus_silerifolius

Ranunculus_taisanensis_2

Ranunculus_taisanensis_3

Raphanus_raphanistrum_subsp._sativus

Rauvolfia_verticillata

Reevesia_formosana

Rhaphiolepis_indica

Rhaphiolepis_indica_var._umbellata

Rhododendron_breviperulatum_1

Rhododendron_breviperulatum_2

Rhododendron_formosanum_1

Rhododendron_formosanum_2

Rhododendron_kanehirai

Rhododendron_latoucheae

Rhododendron_oldhamii_1

Rhododendron_oldhamii_2

Rhododendron_pseudochrysanthum

Rhododendron_rubropilosum_var._taiwanalpinum

Rhododendron_simsii

Rhodomyrtus_tomentosa

Rhynchoglossum_obliquum

Rhynchosia_minima

Ribes_formosanum

Rosa_morrisonensis

Rosa_pricei

Rosa_taiwanensis

Rosa_transmorrisonensis

Rubia_akane_var._erecta

Rubus_corchorifolius_1

Rubus_corchorifolius_2

Rubus_croceacanthus_1

Rubus_croceacanthus_2

Rubus_croceacanthus_3

Rubus_formosensis

Rubus_incanus

Rubus_kawakamii

Rubus_lanyuensis

Rubus_liui

Rubus_pectinellus

Rubus_pentalobus_1

Rubus_pentalobus_2

Rubus_pentalobus_3

Rubus_pungens

Rubus_rosifolius

Rubus_swinhoei

Rubus_taitoensis

Rubus_taiwanicolus_2

Rubus_taiwanicolus_3

Rubus_trianthus

Ruellia_repens

Sagittaria_trifolia

Salvia_arisanensis

Salvia_formosana

Salvia_hayatae

Salvia_nipponica_var._formosana

Sambucus_chinensis_1

Sambucus_chinensis_2

Sarcopyramis_napalensis_var._bodinieri_1

Sarcopyramis_napalensis_var._bodinieri_2

Sarcopyramis_napalensis_var._delicata

Saurauia_tristyla

Saururus_chinensis

Scabiosa_lacerifolia

Scaevola_taccada

Schima_superba

Schisandra_arisanensis

Schizophragma_integrifolium

Scutellaria_indica_1

Scutellaria_indica_2

Scutellaria_tashiroi

Sedum_actinocarpum

Sedum_formosanum

Sedum_morrisonense_1

Sedum_morrisonense_3

Sedum_nokoense

Semecarpus_gigantifolia

Senecio_morrisonensis_var._dentatus_2

Senecio_morrisonensis_var._dentatus_3

Senecio_scandens_var._incisus_2

Senecio_scandens_var._incisus_3

Senecio_scandens_var._scandens

Serissa_japonica

Shortia_rotundifolia

Sibbaldia_procumbens

Sida_insularis

Sida_rhombifolia

Silene_baccifera_2

Silene_baccifera_3

Silene_morrisonmontana_var._glabella

Skimmia_japonica_var._distinctevenulosa

Skimmia_japonica_var._orthoclada

Sloanea_formosana

Smilax_arisanensis

Smilax_china

Smilax_riparia

Solanum_miyakojimense

Solidago_virgaurea

Sophora_tomentosa

Spathoglottis_plicata

Spiraea_formosana_2

Spiraea_formosana_3

Spiraea_hayatana

Spiraea_morrisonicola

Spiraea_prunifolia_var._pseudoprunifolia

Spiranthes_sinensis

Stachyurus_himalaicus

Stauntonia_obovatifoliola_1

Stauntonia_obovatifoliola_2

Stauntonia_purpurea

Staurogyne_concinnula

Stellaria_arisanensis

Stellaria_vestita_2

Stellaria_vestita_3

Sterculia_ceramica

Strobilanthes_flexicaulis

Strobilanthes_formosanus

Strobilanthes_penstemonoides

Strobilanthes_rankanensis

Strobilanthes_wallichii

Styrax_formosanus_1

Styrax_formosanus_2

Styrax_suberifolius

Suzukia_luchuensis

Swertia_macrosperma

Swertia_shintenensis

Swertia_tozanensis

Symplocos_lancifolia

Symplocos_paniculata

Symplocos_sumuntia

Syzygium_tripinnatum

Tabernaemontana_subglobosa

Tarenna_asiatica

Tarennoidea_wallichii

Tephrosia_obovata

Terminalia_catappa

Ternstroemia_gymnanthera

Tetragonia_tetragonioides

Thalictrum_urbainii

Thladiantha_nudiflora

Thladiantha_punctata

Thyrocarpus_sampsonii

Timonius_arboreus

Titanotrichum_oldhamii

Torenia_concolor_1

Torenia_concolor_2

Trachelospermum_asiaticum

Trachelospermum_lanyuense

Tribulus_taiwanense

Trichodesma_calycosum

Trichosanthes_cucumeroides

Trichosanthes_quinquangulata

Trichosanthes_rosthornii

Tricyrtis_formosana_1

Tricyrtis_formosana_2

Tricyrtis_ravenii

Trigonotis_formosana

Trillium_tschonoskii

Triplostegia_glandulifera

Tripterospermum_alutaceifolium

Tripterospermum_lanceolatum

Tripterospermum_luzonense

Tripterospermum_taiwanense

Tristellateia_australasiae

Trochodendron_aralioides

Tylophora_ovata

Uraria_crinita

Urena_lobata

Utricularia_gibba

Utricularia_striatula

Valeriana_fauriei

Valeriana_kawakamii

Veratrum_formosanum

Veratrum_shuehshanarum

Vernicia_montana

Vernonia_maritima

Veronica_morrisonicola

Veronica_oligosperma

Veronica_taiwanica

Viburnum_betulifolium

Viburnum_foetidum_var._rectangulatum

Viburnum_luzonicum

Viburnum_plicatum_var._formosanum

Viburnum_urceolatum

Vigna_marina

Vigna_reflexopilosa

Viola_adenothrix

Viola_adenothrix_var._tsugitakaensis

Viola_arcuata

Viola_betonicifolia

Viola_biflora

Viola_confusa

Viola_formosana_1

Viola_formosana_2

Viola_formosana_var._kawakamii

Viola_grypoceras

Viola_inconspicua_subsp._nagasakiensis

Viola_mandshurica

Viola_nagasawai

Viola_senzanensis

Vitex_negundo

Vitex_trifolia_subsp._litoralis

Volkameria_inermis

Wahlenbergia_marginata

Wedelia_prostrata

Wikstroemia_indica

Wikstroemia_retusa

Zeuxine_affinis

Zeuxine_nervosa

;

END;

BEGIN TREES;

TRANSLATE

1 Abelia_chinensis_var._ionandra,

2 Abutilon_indicum_var._guineense,

3 Acanthephippium_striatum,

4 Acer_caudatifolium,

5 Acer_rubescens,

6 Aconitum_fukutomei,

7 Acronychia_pedunculata,

8 Actinidia_setosa,

9 Adenophora_morrisonensis,

10 Adenophora_morrisonensis_subsp._uehatae_1,

11 Adenophora_morrisonensis_subsp._uehatae_3,

12 Adenophora_triphylla,

13 Aeginetia_indica,

14 Aeschynanthus_acuminatus,

15 Agrimonia_pilosa,

16 Ainsliaea_latifolia_subsp._henryi,

17 Ajuga_taiwanensis,

18 Akebia_longiracemosa,

19 Alangium_chinense,

20 Aletris_spicata,

21 Alniphyllum_pterospermum_1,

22 Alniphyllum_pterospermum_2,

23 Alpinia_flabellata,

24 Alpinia_intermedia,

25 Alpinia_japonica,

26 Alpinia_koshunensis,

27 Alpinia_kusshakuensis,

28 Alpinia_pricei,

29 Alpinia_sessiliflora,

30 Alpinia_shimadae,

31 Alpinia_x_ilanensis,

32 Alpinia_zerumbet,

33 Alysicarpus_vaginalis,

34 Amitostigma_alpestre,

35 Anaphalis_morrisonicola_2,

36 Anaphalis_morrisonicola_3,

37 Anaphalis_nepalensis,

38 Androsace_umbellata,

39 Anemone_vitifolia_2,

40 Anemone_vitifolia_3,

41 Anneslea_fragrans_var._lanceolata,

42 Anodendron_benthamianum,

43 Arabidopsis_lyrata_subsp._kamchatica,

44 Ardisia_cornudentata,

45 Ardisia_cornudentata_subsp._morrisonensis,

46 Ardisia_cornudentata_var._stenosepala,

47 Ardisia_elliptica,

48 Ardisia_humilis,

49 Ardisia_japonica,

50 Ardisia_polysticta,

51 Ardisia_quinquegona,

52 Ardisia_sieboldii,

53 Ardisia_squamulosa,

54 Argostemma_solaniflorum,

55 Arisaema_consanguineum,

56 Arisaema_formosanum_2,

57 Arisaema_heterophyllum,

58 Arisaema_ringens,

59 Aristolochia_cucurbitifolia,

60 Aristolochia_shimadae_1,

61 Aristolochia_shimadae_2,

62 Aristolochia_zollingeriana,

63 Arundina_graminifolia,

64 Asarum_chatienshanianum,

65 Asarum_hypogynum,

66 Asarum_macranthum,

67 Aster_ageratoides,

68 Aster_indicus,

69 Aster_oldhamii,

70 Aster_taiwanensis,

71 Astilbe_longicarpa,

72 Astronia_ferruginea,

73 Aucuba_japonica,

74 Bacopa_monnieri,

75 Balanophora_laxiflora,

76 Barbarea_orthoceras,

77 Barringtonia_racemosa,

78 Barthea_barthei,

79 Bauhinia_championii,

80 Begonia_chitoensis,

81 Begonia_fenicis,

82 Begonia_formosana,

83 Begonia_formosana_2,

84 Begonia_laciniata_1,

85 Begonia_laciniata_2,

86 Begonia_longifolia,

87 Berberis_brevisepala,

88 Berberis_kawakamii,

89 Berberis_mingetsensis,

90 Berberis_morrisonensis,

91 Blastus_cochinchinensis,

92 Bletilla_formosana,

93 Boenninghausenia_albiflora,

94 Bostrychanthera_deflexa,

95 Bredia_oldhamii,

96 Bredia_scandens,

97 Bretschneidera_sinensis,

98 Caesalpinia_crista,

99 Calanthe_aristulifera,

100 Calanthe_puberula,

101 Calanthe_sylvatica,

102 Calanthe_triplicata,

103 Callicarpa_formosana_1,

104 Callicarpa_formosana_2,

105 Callicarpa_japonica_var._luxurians,

106 Callicarpa_kochiana,

107 Callicarpa_pilosissima,

108 Callicarpa_randaiensis,

109 Calophyllum_blancoi,

110 Calophyllum_inophyllum,

111 Camellia_brevistyla,

112 Camellia_euryoides_var._nokoensis,

113 Camellia_furfuracea,

114 Camellia_hengchunensis,

115 Camellia_japonica,

116 Camellia_tenuiflora,

117 Canavalia_lineata,

118 Canavalia_rosea,

119 Cardiandra_formosana,

120 Carpesium_nepalense_2,

121 Carpesium_nepalense_3,

122 Celastrus_punctatus,

123 Centaurium_japonicum,

124 Cephalanthera_alpicola,

125 Cephalantheropsis_obcordata,

126 Cerastium_morrisonense_2,

127 Cerastium_morrisonense_3,

128 Cerbera_manghas,

129 Chamaecrista_garambiensis,

130 Cheilotheca_humilis_2,

131 Cheilotheca_humilis_3,

132 Cheilotheca_macrocarpa,

133 Cheirostylis_octodactyla,

134 Chimaphila_japonica,

135 Chionanthus_ramiflorus,

136 Chionanthus_retusus,

137 Cirsium_brevicaule,

138 Cirsium_japonicum_var._australe,

139 Cirsium_suzukii,

140 Citrus_reticulata_var._depressa,

141 Clematis_chinensis,

142 Clematis_grata_1,

143 Clematis_grata_2,

144 Clematis_lasiandra,

145 Clematis_montana,

146 Clematis_tashiroi,

147 Clematis_tsugetorum,

148 Clerodendrum_cyrtophyllum,

149 Clerodendrum_japonicum,

150 Clerodendrum_trichotomum_1,

151 Clerodendrum_trichotomum_2,

152 Clinopodium_chinense_1,

153 Clinopodium_chinense_2,

154 Clinopodium_chinense_3,

155 Clinopodium_gracile,

156 Clinopodium_laxiflorum,

157 Clinopodium_laxiflorum_var._taiwanianum,

158 Codonacanthus_pauciflorus,

159 Codonopsis_kawakamii,

160 Coleus_formosanus,

161 Commelina_auriculata,

162 Commelina_communis,

163 Commelina_diffusa,

164 Conandron_ramondioides,

165 Coptis_quinquefolia,

166 Corydalis_ochotensis,

167 Corydalis_ophiocarpa,

168 Corydalis_pallida,

169 Corydalis_tashiroi_1,

170 Corydalis_tashiroi_2,

171 Cotoneaster_konishii,

172 Cotoneaster_morrisonensis,

173 Cremastra_appendiculata_var._variabilis,

174 Crepidiastrum_taiwanianum,

175 Crepidium_matsudae,

176 Crossostephium_chinense,

177 Crotalaria_similis,

178 Cymbidium_ensifolium,

179 Cymbidium_sinense,

180 Cynanchum_lanhsuense,

181 Cynanchum_ovalifolium,

182 Cynoglossum_alpestre,

183 Cynoglossum_furcatum,

184 Cypripedium_debile,

185 Damnacanthus_indicus,

186 Daphne_arisanensis_2,

187 Daphne_arisanensis_3,

188 Daphne_kiusiana_var._atrocaulis,

189 Decaspermum_gracilentum,

190 Dendrobium_catenatum,

191 Dendrobium_goldschmidtianum,

192 Dendrolobium_umbellatum,

193 Derris_oblonga,

194 Deutzia_pulchra_1,

195 Deutzia_pulchra_2,

196 Deutzia_pulchra_3,

197 Deutzia_taiwanensis,

198 Dianella_ensifolia,

199 Dianthus_pygmaeus_2,

200 Dianthus_pygmaeus_3,

201 Dianthus_superbus_subsp._longicalycinus,

202 Diospyros_discolor,

203 Diospyros_maritima,

204 Disporum_nantouense,

205 Disporum_sessile,

206 Donax_canniformis,

207 Ehretia_philippinensis,

208 Elaeagnus_formosensis,

209 Elaeagnus_umbellata,

210 Elaeocarpus_argenteus,

211 Elaeocarpus_japonicus,

212 Ellisiophyllum_pinnatum_2,

213 Ellisiophyllum_pinnatum_3,

214 Embelia_laeta_subsp._papilligera,

215 Enkianthus_perulatus,

216 Epilobium_amurense_2,

217 Epilobium_amurense_3,

218 Epilobium_brevifolium_subsp._trichoneurum,

219 Epilobium_hohuanense,

220 Erigeron_fukuyamae,

221 Erigeron_morrisonensis,

222 Eriobotrya_deflexa_f._koshunensis,

223 Erythrina_variegata,

224 Eulophia_pulchra,

225 Eulophia_pulchra_var._actinomorpha,

226 Euonymus_cochinchinensis,

227 Euonymus_japonicus,

228 Euphorbia_jolkinii,

229 Euphrasia_transmorrisonensis,

230 Eurya_chinensis,

231 Evolvulus_alsinoides,

232 Farfugium_japonicum,

233 Filipendula_kiraishiensis,

234 Fissistigma_glaucescens,

235 Flacourtia_rukam,

236 Fragaria_hayatai,

237 Galactia_tashiroi,

238 Galeola_falconeri,

239 Garcinia_multiflora,

240 Garcinia_subelliptica,

241 Gardenia_jasminoides,

242 Gaultheria_borneensis_2,

243 Gaultheria_borneensis_3,

244 Gaultheria_leucocarpa_var._cumingiana,

245 Geniostoma_rupestre,

246 Gentiana_arisanensis,

247 Gentiana_davidii_var._formosana_2,

248 Gentiana_davidii_var._formosana_3,

249 Gentiana_flavomaculata_2,

250 Gentiana_flavomaculata_3,

251 Gentiana_flavomaculata_var._yuanyanghuensis,

252 Gentiana_scabrida_var._punctulata,

253 Geophila_repens,

254 Geranium_hayatanum,

255 Geranium_robertianum,

256 Geranium_suzukii,

257 Geranium_thunbergii,

258 Geranium_wilfordii,

259 Glechoma_hederacea,

260 Glochidion_lanceolatum,

261 Glochidion_rubrum,

262 Glossocardia_bidens,

263 Goniothalamus_amuyon,

264 Goodyera_foliosa,

265 Goodyera_nankoensis,

266 Goodyera_procera,

267 Goodyera_rubicunda,

268 Gordonia_axillaris,

269 Guettarda_speciosa,

270 Gymnema_sylvestre,

271 Gymnosporia_emarginata,

272 Gynura_elliptica,

273 Hedyotis_strigulosa_var._parvifolia,

274 Heliotropium_indicum,

275 Heloniopsis_umbellata_1,

276 Heloniopsis_umbellata_2,

277 Helwingia_japonica_subsp._formosana,

278 Hemiboea_bicornuta_1,

279 Hemiboea_bicornuta_2,

280 Hemigraphis_reptans,

281 Hemiphragma_heterophyllum,

282 Heritiera_littoralis,

283 Hernandia_nymphaeifolia,

284 Hewittia_malabarica,

285 Hibiscus_mutabilis,

286 Hibiscus_taiwanensis_1,

287 Hibiscus_taiwanensis_2,

288 Hibiscus_tilliaceus,

289 Hieracium_morii,

290 Houttuynia_cordata_1,

291 Houttuynia_cordata_2,

292 Hybanthus_enneaspermus,

293 Hydrangea_angustipetala,

294 Hydrangea_aspera,

295 Hydrangea_chinensis_1,

296 Hydrangea_chinensis_2,

297 Hydrangea_integrifolia,

298 Hygrophila_pogonocalyx,

299 Hylodesmum_leptopus,

300 Hypericum_formosanum_1,

301 Hypericum_formosanum_2,

302 Hypericum_nagasawae,

303 Hypericum_subalatum,

304 Hypericum_taihezanense,

305 Hypoestes_cumingiana,

306 Hypoestes_purpurea,

307 Ilex_asprella,

308 Ilex_ficoidea,

309 Ilex_kusanoi,

310 Ilex_maximowicziana,

311 Illicium_anisatum,

312 Illicium_arborescens,

313 Impatiens_tayemonii,

314 Impatiens_uniflora_2,

315 Impatiens_uniflora_3,

316 Indigofera_byobiensis,

317 Indigofera_pedicellata,

318 Indigofera_ramulosissima,

319 Indigofera_spicata,

320 Indigofera_trifoliata,

321 Indigofera_zollingeriana,

322 Ipomoea_imperati,

323 Ipomoea_littoralis,

324 Ipomoea_pes-caprae_subsp._brasiliensis,

325 Iris_domestica,

326 Itea_parviflora,

327 Ixeridium_laevigatum,

328 Ixeris_chinensis,

329 Jasminanthes_mucronata,

330 Jasminum_nervosum,

331 Justicia_procumbens_1,

332 Justicia_procumbens_2,

333 Justicia_procumbens_var._hayatae,

334 Kalanchoe_garambiensis,

335 Kalanchoe_integra,

336 Kleinhovia_hospita,

337 Koelreuteria_elegans_subsp._formosana,

338 Lagerstroemia_subcostata,

339 Lasianthus_attenuatus,

340 Lasianthus_fordii,

341 Lathraea_purpurea,

342 Leea_guineensis,

343 Leea_philippinensis,

344 Leontopodium_microphyllum,

345 Leonurus_japonicus,

346 Lespedeza_formosa,

347 Leucas_chinensis,

348 Ligularia_kojimae,

349 Ligustrum_liukiuense,

350 Ligustrum_sinense,

351 Lilium_longiflorum_var._formosanum_1,

352 Lilium_longiflorum_var._formosanum_3,

353 Lilium_longiflorum_var._scabrum,

354 Limnophila_aromatica,

355 Lindernia_ruellioides,

356 Liparis_bootanensis,

357 Liparis_formosana,

358 Liparis_gigantea,

359 Liparis_henryi,

360 Liparis_sootenzanensis,

361 Liriope_muscari,

362 Litsea_cubeba,

363 Lobelia_nummularia_1,

364 Lobelia_nummularia_2,

365 Lobelia_seguinii,

366 Lonicera_acuminata_2,

367 Lonicera_acuminata_3,

368 Lonicera_apodantha,

369 Lonicera_japonica_1,

370 Lonicera_japonica_2,

371 Ludwigia_octovalvis,

372 Lycianthes_biflora,

373 Lycianthes_lysimachioides,

374 Lysimachia_ardisioides_1,

375 Lysimachia_ardisioides_2,

376 Lysimachia_congestiflora,

377 Lysimachia_mauritiana,

378 Lysimachia_remota,

379 Lysionotus_pauciflorus_1,

380 Lysionotus_pauciflorus_2,

381 Maesa_japonica,

382 Maesa_lanyuensis,

383 Maianthemum_formosanum,

384 Marsdenia_formosana,

385 Mazus_alpinus_2,

386 Mazus_alpinus_3,

387 Mazus_fauriei,

388 Mazus_goodenifolius,

389 Medinilla_fengii_1,

390 Medinilla_fengii_2,

391 Medinilla_formosana,

392 Melanolepis_multiglandulosa,

393 Melastoma_malabathricum,

394 Melicope_triphylla,

395 Melodinus_angustifolius,

396 Memecylon_lanceolatum,

397 Merremia_gemella,

398 Michelia_compressa_var._formosana,

399 Michelia_compressa_var._lanyuensis,

400 Millettia_pulchra_var._microphylla,

401 Millettia_reticulata,

402 Molineria_capitulata,

403 Moneses_uniflora,

404 Monotropa_hypopitys,

405 Morinda_citrifolia,

406 Mucuna_macrocarpa,

407 Mucuna_membranacea,

408 Murdannia_loriformis,

409 Murraya_crenulata,

410 Murraya_paniculata_var._omphalocarpa,

411 Mussaenda_formosanum,

412 Mussaenda_macrophylla,

413 Mussaenda_parviflora,

414 Mussaenda_taihokuensis_1,

415 Mussaenda_taihokuensis_2,

416 Myristica_ceylanica,

417 Myristica_elliptica_var._simiarum,

418 Myrmechis_drymoglossifolia,

419 Neanotis_formosana,

420 Nemosenecio_formosanus,

421 Neottia_formosana,

422 Neottia_meifongensis,

423 Nothapodytes_nimmoniana,

424 Odontochilus_bisaccatus,

425 Olax_imbricata,

426 Ophiopogon_intermedius_1,

427 Ophiopogon_intermedius_2,

428 Ophiopogon_intermedius_3,

429 Ophiorrhiza_japonica,

430 Ophiorrhiza_kuroiwae,

431 Ophiorrhiza_pumila,

432 Origanum_vulgare_1,

433 Origanum_vulgare_3,

434 Osteomeles_anthyllidifolia,

435 Oxalis_acetosella_2,

436 Oxalis_acetosella_3,

437 Palaquium_formosanum,

438 Paraphlomis_javanica,

439 Paraprenanthes_sororia,

440 Paris_polyphylla_var._stenophylla_2,

441 Paris_polyphylla_var._stenophylla_3,

442 Parnassia_palustris_2,

443 Parnassia_palustris_3,

444 Parsonsia_alboflavescens,

445 Patrinia_glabrifolia,

446 Pedicularis_verticillata,

447 Pemphis_acidula,

448 Pentacoelium_bontioides,

449 Peracarpa_carnosa_2,

450 Peracarpa_carnosa_3,

451 Peristrophe_bivalvis,

452 Peristrophe_japonica,

453 Persicaria_chinensis,

454 Petasites_formosanus_2,

455 Petasites_formosanus_3,

456 Phaius_mishmensis,

457 Phaius_tankervilleae,

458 Philydrum_lanuginosum,

459 Photinia_beauverdiana,

460 Photinia_lucida,

461 Photinia_niitakayamensis,

462 Picris_morrisonensis_2,

463 Picris_morrisonensis_3,

464 Pieris_japonica,

465 Pittosporum_illicioides,

466 Pittosporum_pentandrum,

467 Platanthera_brevicalcarata,

468 Platanthera_mandarinorum_subsp._pachyglossa_2,

469 Platanthera_mandarinorum_subsp._pachyglossa_3,

470 Platanthera_sachalinensis,

471 Polygala_sibirica,

472 Polygonatum_arisanense_1,

473 Polygonatum_arisanense_2,

474 Polygonum_biconvexum,

475 Polygonum_cuspidatum_2,

476 Polygonum_cuspidatum_3,

477 Polygonum_posumbu,

478 Polygonum_runcinatum,

479 Pongamia_pinnata,

480 Portulaca_oleracea,

481 Portulaca_pilosa,

482 Potentilla_leuconota,

483 Potentilla_matsumurae_var._pilosa,

484 Premna_hengchunensis,

485 Premna_microphylla,

486 Premna_serratifolia,

487 Primula_miyabeana,

488 Prinsepia_scandens,

489 Prunella_vulgaris_subsp._asiatica_1,

490 Prunella_vulgaris_subsp._asiatica_2,

491 Prunella_vulgaris_var._nanhutashanense,

492 Prunus_campanulata,

493 Prunus_phaeosticta,

494 Prunus_salicina,

495 Prunus_transarisanensis,

496 Pueraria_montana_1,

497 Pueraria_montana_2,

498 Pyracantha_koidzumii_1,

499 Pyracantha_koidzumii_2,

500 Pyrola_morrisonensis,

501 Radermachera_sinica,

502 Ranunculus_cantoniensis_1,

503 Ranunculus_cantoniensis_2,

504 Ranunculus_formosa-montanus,

505 Ranunculus_junipericola,

506 Ranunculus_sieboldii,

507 Ranunculus_silerifolius,

508 Ranunculus_taisanensis_2,

509 Ranunculus_taisanensis_3,

510 Raphanus_raphanistrum_subsp._sativus,

511 Rauvolfia_verticillata,

512 Reevesia_formosana,

513 Rhaphiolepis_indica,

514 Rhaphiolepis_indica_var._umbellata,

515 Rhododendron_breviperulatum_1,

516 Rhododendron_breviperulatum_2,

517 Rhododendron_formosanum_1,

518 Rhododendron_formosanum_2,

519 Rhododendron_kanehirai,

520 Rhododendron_latoucheae,

521 Rhododendron_oldhamii_1,

522 Rhododendron_oldhamii_2,

523 Rhododendron_pseudochrysanthum,

524 Rhododendron_rubropilosum_var._taiwanalpinum,

525 Rhododendron_simsii,

526 Rhodomyrtus_tomentosa,

527 Rhynchoglossum_obliquum,

528 Rhynchosia_minima,

529 Ribes_formosanum,

530 Rosa_morrisonensis,

531 Rosa_pricei,

532 Rosa_taiwanensis,

533 Rosa_transmorrisonensis,

534 Rubia_akane_var._erecta,

535 Rubus_corchorifolius_1,

536 Rubus_corchorifolius_2,

537 Rubus_croceacanthus_1,

538 Rubus_croceacanthus_2,

539 Rubus_croceacanthus_3,

540 Rubus_formosensis,

541 Rubus_incanus,

542 Rubus_kawakamii,

543 Rubus_lanyuensis,

544 Rubus_liui,

545 Rubus_pectinellus,

546 Rubus_pentalobus_1,

547 Rubus_pentalobus_2,

548 Rubus_pentalobus_3,

549 Rubus_pungens,

550 Rubus_rosifolius,

551 Rubus_swinhoei,

552 Rubus_taitoensis,

553 Rubus_taiwanicolus_2,

554 Rubus_taiwanicolus_3,

555 Rubus_trianthus,

556 Ruellia_repens,

557 Sagittaria_trifolia,

558 Salvia_arisanensis,

559 Salvia_formosana,

560 Salvia_hayatae,

561 Salvia_nipponica_var._formosana,

562 Sambucus_chinensis_1,

563 Sambucus_chinensis_2,

564 Sarcopyramis_napalensis_var._bodinieri_1,

565 Sarcopyramis_napalensis_var._bodinieri_2,

566 Sarcopyramis_napalensis_var._delicata,

567 Saurauia_tristyla,

568 Saururus_chinensis,

569 Scabiosa_lacerifolia,

570 Scaevola_taccada,

571 Schima_superba,

572 Schisandra_arisanensis,

573 Schizophragma_integrifolium,

574 Scutellaria_indica_1,

575 Scutellaria_indica_2,

576 Scutellaria_tashiroi,

577 Sedum_actinocarpum,

578 Sedum_formosanum,

579 Sedum_morrisonense_1,

580 Sedum_morrisonense_3,

581 Sedum_nokoense,

582 Semecarpus_gigantifolia,

583 Senecio_morrisonensis_var._dentatus_2,

584 Senecio_morrisonensis_var._dentatus_3,

585 Senecio_scandens_var._incisus_2,

586 Senecio_scandens_var._incisus_3,

587 Senecio_scandens_var._scandens,

588 Serissa_japonica,

589 Shortia_rotundifolia,

590 Sibbaldia_procumbens,

591 Sida_insularis,

592 Sida_rhombifolia,

593 Silene_baccifera_2,

594 Silene_baccifera_3,

595 Silene_morrisonmontana_var._glabella,

596 Skimmia_japonica_var._distinctevenulosa,

597 Skimmia_japonica_var._orthoclada,

598 Sloanea_formosana,

599 Smilax_arisanensis,

600 Smilax_china,

601 Smilax_riparia,

602 Solanum_miyakojimense,

603 Solidago_virgaurea,

604 Sophora_tomentosa,

605 Spathoglottis_plicata,

606 Spiraea_formosana_2,

607 Spiraea_formosana_3,

608 Spiraea_hayatana,

609 Spiraea_morrisonicola,

610 Spiraea_prunifolia_var._pseudoprunifolia,

611 Spiranthes_sinensis,

612 Stachyurus_himalaicus,

613 Stauntonia_obovatifoliola_1,

614 Stauntonia_obovatifoliola_2,

615 Stauntonia_purpurea,

616 Staurogyne_concinnula,

617 Stellaria_arisanensis,

618 Stellaria_vestita_2,

619 Stellaria_vestita_3,

620 Sterculia_ceramica,

621 Strobilanthes_flexicaulis,

622 Strobilanthes_formosanus,

623 Strobilanthes_penstemonoides,

624 Strobilanthes_rankanensis,

625 Strobilanthes_wallichii,

626 Styrax_formosanus_1,

627 Styrax_formosanus_2,

628 Styrax_suberifolius,

629 Suzukia_luchuensis,

630 Swertia_macrosperma,

631 Swertia_shintenensis,

632 Swertia_tozanensis,

633 Symplocos_lancifolia,

634 Symplocos_paniculata,

635 Symplocos_sumuntia,

636 Syzygium_tripinnatum,

637 Tabernaemontana_subglobosa,

638 Tarenna_asiatica,

639 Tarennoidea_wallichii,

640 Tephrosia_obovata,

641 Terminalia_catappa,

642 Ternstroemia_gymnanthera,

643 Tetragonia_tetragonioides,

644 Thalictrum_urbainii,

645 Thladiantha_nudiflora,

646 Thladiantha_punctata,

647 Thyrocarpus_sampsonii,

648 Timonius_arboreus,

649 Titanotrichum_oldhamii,

650 Torenia_concolor_1,

651 Torenia_concolor_2,

652 Trachelospermum_asiaticum,

653 Trachelospermum_lanyuense,

654 Tribulus_taiwanense,

655 Trichodesma_calycosum,

656 Trichosanthes_cucumeroides,

657 Trichosanthes_quinquangulata,

658 Trichosanthes_rosthornii,

659 Tricyrtis_formosana_1,

660 Tricyrtis_formosana_2,

661 Tricyrtis_ravenii,

662 Trigonotis_formosana,

663 Trillium_tschonoskii,

664 Triplostegia_glandulifera,

665 Tripterospermum_alutaceifolium,

666 Tripterospermum_lanceolatum,

667 Tripterospermum_luzonense,

668 Tripterospermum_taiwanense,

669 Tristellateia_australasiae,

670 Trochodendron_aralioides,

671 Tylophora_ovata,

672 Uraria_crinita,

673 Urena_lobata,

674 Utricularia_gibba,

675 Utricularia_striatula,

676 Valeriana_fauriei,

677 Valeriana_kawakamii,

678 Veratrum_formosanum,

679 Veratrum_shuehshanarum,

680 Vernicia_montana,

681 Vernonia_maritima,

682 Veronica_morrisonicola,

683 Veronica_oligosperma,

684 Veronica_taiwanica,

685 Viburnum_betulifolium,

686 Viburnum_foetidum_var._rectangulatum,

687 Viburnum_luzonicum,

688 Viburnum_plicatum_var._formosanum,

689 Viburnum_urceolatum,

690 Vigna_marina,

691 Vigna_reflexopilosa,

692 Viola_adenothrix,

693 Viola_adenothrix_var._tsugitakaensis,

694 Viola_arcuata,

695 Viola_betonicifolia,

696 Viola_biflora,

697 Viola_confusa,

698 Viola_formosana_1,

699 Viola_formosana_2,

700 Viola_formosana_var._kawakamii,

701 Viola_grypoceras,

702 Viola_inconspicua_subsp._nagasakiensis,

703 Viola_mandshurica,

704 Viola_nagasawai,

705 Viola_senzanensis,

706 Vitex_negundo,

707 Vitex_trifolia_subsp._litoralis,

708 Volkameria_inermis,

709 Wahlenbergia_marginata,

710 Wedelia_prostrata,

711 Wikstroemia_indica,

712 Wikstroemia_retusa,

713 Zeuxine_affinis,

714 Zeuxine_nervosa

;

TREE * UNTITLED = [&R] (((((((((((((((((((((((278:18.6973,279:18.6973)737:18.6973,14:37.3946,(379:18.6973,380:18.6973)738:18.6973,164:37.3946,527:37.3946,649:37.3946)736:3.57298,448:40.9676)735:0.094686,((387:13.7031,388:13.7031,385:13.7031,386:13.7031)740:24.4297,501:38.1329)739:2.92938)734:0.332342,((561:16.4421,558:16.4421,559:16.4421,560:16.4421)742:16.4421,(156:16.4421,157:16.4421,153:16.4421,155:16.4421,152:16.4421,154:16.4421)743:16.4421,17:32.8843,(433:16.4421,432:16.4421)744:16.4421,347:32.8843,708:32.8843,(150:16.4421,151:16.4421)745:16.4421,(106:16.4421,107:16.4421,103:16.4421,104:16.4421,108:16.4421,105:16.4421)746:16.4421,707:32.8843,629:32.8843,(490:16.4421,489:16.4421,491:16.4421)747:16.4421,(574:16.4421,575:16.4421,576:16.4421)748:16.4421,160:32.8843,484:32.8843,94:32.8843,((485:13.7952,((149:1.64822,148:1.64822)752:11.1618,(345:4.31199,438:4.31199)753:8.498)751:0.985182)750:8.6915,(706:17.4243,(486:16.2893,259:16.2893)755:1.135)754:5.0624)749:10.3976)741:8.51031)733:0.621584,(158:36.721,280:36.721,298:36.721,(305:18.3605,306:18.3605)757:18.3605,(331:18.3605,333:18.3605,332:18.3605)758:18.3605,(451:18.3605,452:18.3605)759:18.3605,556:36.721,616:36.721,(624:18.3605,625:18.3605,622:18.3605,621:18.3605,623:18.3605)760:18.3605)756:5.29517)732:0.129732,(13:35.494,341:35.494,229:35.494,446:35.494)761:6.65187)731:0.182246,(675:39.9512,674:39.9512)762:2.37694)730:0.432809,(((650:10.2929,651:10.2929)765:10.2929,355:20.5859)764:12.3366,((684:15.854,683:15.854)767:15.854,(212:15.854,213:15.854)768:15.854,((74:12.9665,354:12.9665)770:9.86285,(281:9.38628,682:9.38628)771:13.4431)769:8.87857)766:1.21457)763:9.83844)729:13.4796,(330:39.3526,349:39.3526,((135:1.12368,136:1.12368)774:21.6273,350:22.751)773:16.6016)772:16.8879)728:16.5865,((373:47.0276,602:47.0276,372:47.0276)776:9.21285,((324:27.0735,323:27.0735)778:27.0735,397:54.147,284:54.147,(231:16.1045,322:16.1045)779:38.0425)777:2.0935)775:16.5865)727:0.542387,(((((181:18.8041,180:18.8041)784:18.8041,270:37.6083,329:37.6083,395:37.6083,444:37.6083,511:37.6083,637:37.6083,653:37.6083,671:37.6083,42:37.6083,384:37.6083,(128:18.6328,652:18.6328)785:18.9754)783:9.14693,245:46.7552)782:5.27365,((246:14.8236,249:14.8236,252:14.8236,250:14.8236,247:14.8236,248:14.8236,251:14.8236)787:14.8236,(632:14.8236,631:14.8236)788:14.8236,123:29.6472,(668:14.8236,666:14.8236,667:14.8236,665:14.8236)789:14.8236,630:29.6472)786:22.3816)781:6.77608,(638:56.8699,648:56.8699,419:56.8699,(340:28.435,339:28.435)791:28.435,(414:28.435,413:28.435,411:28.435,415:28.435,412:28.435)792:28.435,534:56.8699,639:56.8699,54:56.8699,273:56.8699,((((430:0.540847,431:0.540847)796:2.22283,429:2.76368)795:34.2895,(((185:25.7114,405:25.7114)799:0.744165,253:26.4556)798:6.14223,588:32.5978)797:4.45537)794:10.5937,(269:33.1855,241:33.1855)800:14.4613)793:9.22313)790:1.93497)780:14.5645)726:1.62956,(655:55.7952,662:55.7952,207:55.7952,(183:27.8976,182:27.8976)802:27.8976,647:55.7952,274:55.7952)801:19.2038)725:4.13767,423:79.1367)724:17.8136,73:96.9503)723:5.92122,(((((685:37.6926,686:37.6926,688:37.6926,687:37.6926)807:37.6926,(562:16.845,563:16.845)808:58.5401,689:75.3851)806:15.9708,((367:32.8016,366:32.8016,370:32.8016,369:32.8016,368:32.8016)810:32.8016,569:65.6031,445:65.6031,1:65.6031,677:65.6031,(664:46.4875,676:46.4875)811:19.1157)809:25.7528)805:4.96097,((465:56.7779,466:56.7779)813:38.6389,(((363:26.4836,364:26.4836,365:26.4836)816:26.4836,(9:26.4836,11:26.4836,10:26.4836)817:26.4836,(449:26.4836,450:26.4836)818:26.4836,159:52.9672,709:52.9672,12:52.9672)815:30.6964,(((37:22.8719,36:22.8719,35:22.8719)821:22.8719,439:45.7437,(584:22.8719,587:22.8719,585:22.8719,583:22.8719,586:22.8719)822:22.8719,(137:22.8719,139:22.8719,138:22.8719)823:22.8719,681:45.7437,(70:22.8719,69:22.8719)824:22.8719,(121:22.8719,120:22.8719)825:22.8719,327:45.7437,(454:22.8719,455:22.8719)826:22.8719,174:45.7437,262:45.7437,272:45.7437,328:45.7437,(462:22.8719,463:22.8719)827:22.8719,16:45.7437,289:45.7437,176:45.7437,(221:22.8719,220:22.8719)828:22.8719,348:45.7437,420:45.7437,((232:24.284,((67:1.49361,68:1.49361)832:6.13541,603:7.62902)831:16.6549)830:3.98855,(344:24.5937,710:24.5937)833:3.67877)829:17.4712)820:10.4505,570:56.1942)819:27.4695)814:11.7531)812:0.900124)804:4.67238,(277:69.0702,(309:65.833,((310:11.4515,308:11.4515)837:6.18518,307:17.6367)836:48.1964)835:3.23717)834:31.9191)803:1.88221)722:4.73798,(((((((41:66.0501,(642:43.8733,230:43.8733)845:22.1768)844:5.7751,(((112:29.2324,114:29.2324,116:29.2324)848:29.2324,268:58.4648,(571:57.076,(115:13.0553,(113:3.59122,111:3.59122)851:9.46413)850:44.0207)849:1.38881)847:9.56574,437:68.0306)846:3.7946)843:7.81911,(203:8.74969,202:8.74969)852:70.8946)842:3.12388,((((244:31.0258,242:31.0258,243:31.0258)856:31.0258,134:62.0517,215:62.0517,(518:31.0258,522:31.0258,515:31.0258,516:31.0258,521:31.0258,517:31.0258,524:31.0258,520:31.0258)857:31.0258,(132:31.0258,130:31.0258,131:31.0258)858:31.0258,500:62.0517,(404:53.6508,(403:50.5009,(((525:1.41887,519:1.41887)863:8.0396,523:9.45846)862:34.0454,464:43.5038)861:6.99707)860:3.14994)859:8.40083)855:16.7656,((22:31.987,21:31.987)865:31.987,(626:31.987,627:31.987)866:31.987,628:63.9739)864:14.8433)854:3.36832,((567:61.4993,8:61.4993)868:15.6753,((634:40.8712,(635:27.1983,633:27.1983)871:13.6729)870:34.6304,77:75.50154)869:1.673)867:5.011)853:0.582584)841:2.80459,(((50:27.0199,52:27.0199,45:27.0199,48:27.0199,53:27.0199,44:27.0199,46:27.0199)874:27.0199,214:54.0398,38:54.0398,(378:27.0199,375:27.0199,374:27.0199)875:27.0199,382:54.0398,381:54.0398,((((51:1.92136,47:1.92136)879:2.66447,49:4.58584)878:24.593,(377:11.2789,376:11.2789)880:17.8999)877:5.86275,487:35.0415)876:18.9983)873:18.4887,589:72.5285)872:13.0442)840:6.87514,(315:20.9198,313:20.9198,314:20.9198)881:71.5281)839:13.2279,(((195:31.8948,197:31.8948,194:31.8948,196:31.8948)884:31.8948,(297:31.8948,293:31.8948,295:31.8948,296:31.8948)885:31.8948,573:63.7896,(119:22.0066,294:22.0066)886:41.783)883:33.9508,19:97.7405)882:7.93534)838:1.93366)721:9.08781,((425:60.9815,75:60.9815)888:54.1795,(((477:27.0072,475:27.0072,474:27.0072,476:27.0072)891:27.0072,453:54.0144,478:54.0144)890:35.4465,(((126:25.3025,127:25.3025)894:25.3025,(593:25.3025,594:25.3025,595:25.3025)895:25.3025,(201:25.3025,200:25.3025,199:25.3025)896:25.3025,(619:25.3025,617:25.3025,618:25.3025)897:25.3025)893:10.3953,(643:56.719,(480:16.493,481:16.493)899:40.226)898:4.28119)892:28.4607)889:25.7001)887:1.53628)720:2.46823,(((((((((582:70.9407,(((410:22.7366,409:22.7366)911:22.7366,(597:22.7366,596:22.7366)912:22.7366,140:45.4732,(93:28.5711,(7:3.74711,394:3.74711)914:24.824)913:16.9021)910:23.1894,(5:66.2502,337:66.2502,4:66.2502)915:2.41233)909:2.27819)908:29.5729,(((285:34.5597,286:34.5597,288:34.5597,287:34.5597)918:34.5597,512:69.1194,2:69.1194,620:69.1194,591:69.1194,(336:56.8537,(282:32.7366,(673:19.7791,592:19.7791)921:12.9576)920:24.1171)919:12.2657)917:11.8802,((186:29.346,188:29.346,187:29.346)923:29.346,712:58.6919,711:58.6919)922:22.3076)916:19.5141)907:0.908691,((510:60.7464,43:60.7464,76:60.7464)925:16.5726,97:77.3189)924:24.1034)906:9.59952,(((((189:71.3021,636:71.3021,526:71.3021)930:11.5898,((96:29.9217,95:29.9217)932:29.9217,78:59.8433,72:59.8433,(566:29.9217,564:29.9217,565:29.9217)933:29.9217,(390:29.9217,391:29.9217,389:29.9217)934:29.9217,396:59.8433,(393:27.4839,91:27.4839)935:32.3594)931:23.0485)929:3.60106,(338:67.9761,447:67.9761)936:18.5168)928:0.728903,641:87.2218)927:0.978722,((216:31.262,217:31.262,219:31.262,218:31.262)938:31.262,371:62.524)937:25.6765)926:22.8213)905:1.64657,612:112.668)904:0.58204,((256:36.8672,254:36.8672)940:36.8672,(255:34.6583,(258:3.37788,257:3.37788)942:31.2804)941:39.076)939:39.5161)903:3.62701,(343:25.5532,342:25.5532)943:91.3243)902:0.657279,(654:113.68,((((((85:18.4982,82:18.4982,84:18.4982,80:18.4982,81:18.4982)950:18.4982,(86:3.57919,83:3.57919)951:33.4173)949:36.3884,((657:34.1784,656:34.1784,658:34.1784)953:34.1784,(646:34.1784,645:34.1784)954:34.1784)952:5.02815)948:32.5511,(((531:41.147,530:41.147,532:41.147,533:41.147)957:41.147,(535:41.147,553:41.147,536:41.147,544:41.147,548:41.147,546:41.147,542:41.147,538:41.147,554:41.147,547:41.147,552:41.147,540:41.147,543:41.147,539:41.147,549:41.147,541:41.147,545:41.147,537:41.147)958:41.147,(492:41.147,495:41.147)959:41.147,222:82.294,(499:41.147,498:41.147)960:41.147,171:82.294,514:82.294,(460:41.147,461:41.147)961:41.147,(482:41.147,483:41.147)962:41.147,(606:41.147,610:41.147,609:41.147,607:41.147,608:41.147)963:41.147,236:82.294,233:82.294,488:82.294,((551:28.8287,(555:8.15797,550:8.15797)966:20.6707)965:31.3555,(15:55.964,590:55.964)967:4.22017)964:22.1099,((493:23.2159,494:23.2159)969:3.01031,(459:4.42304,(434:3.70536,(172:2.4212,513:2.4212)972:1.28416)971:0.717685)970:21.8032)968:56.0678)956:6.98304,(208:56.4399,209:56.4399)973:32.8372)955:16.6589)947:1.05941,((223:69.1425,299:69.1425,672:69.1425,33:69.1425,129:69.1425,691:69.1425,98:69.1425,(321:34.5713,320:34.5713,316:34.5713,317:34.5713,318:34.5713)976:34.5713,479:69.1425,(497:34.5713,496:34.5713)977:34.5713,177:69.1425,117:69.1425,192:69.1425,193:69.1425,640:69.1425,407:69.1425,346:69.1425,(401:34.5713,400:34.5713)978:34.5713,79:69.1425,(604:53.0735,(319:41.1329,((118:18.6939,237:18.6939)982:16.3982,(406:21.2758,(528:9.59623,690:9.59623)984:11.6796)983:13.8163)981:6.04084)980:11.9406)979:16.069)975:2.90626,471:72.0488)974:34.9466)946:6.26322,((((436:28.2296,435:28.2296)988:59.8195,(598:70.1596,210:70.1596,211:70.1596)989:17.8896)987:23.3319,((260:98.3195,261:98.3195)991:5.9755,((((228:99.388,(392:70.9646,680:70.9646)996:28.4234)995:0.597179,669:99.9852)994:0.59605,(((704:29.5247,698:29.5247,692:29.5247,700:29.5247,702:29.5247,695:29.5247,699:29.5247,705:29.5247,697:29.5247,693:29.5247)999:29.5247,(292:38.891,(701:14.524,(696:12.4812,(694:4.35982,703:4.35982)1003:8.12134)1002:2.04281)1001:24.367)1000:20.1585)998:37.8502,235:96.8997)997:3.68157)993:0.532812,(((304:28.537,303:28.537,301:28.537,302:28.537,300:28.537)1006:42.648,(239:68.7467,240:68.7467)1007:2.43827)1005:15.087,(109:82.9693,110:82.9693)1008:3.30263)1004:14.8421)992:3.18096)990:7.08601)986:0.109862,(226:67.7436,271:67.7436,122:67.7436,(442:33.8718,443:33.8718)1010:33.8718,227:67.7436)1009:43.7473)985:1.76773)945:0.420986)944:3.85514)901:0.138911,(((577:35.134,579:35.134,580:35.134,581:35.134,578:35.134)1013:35.134,(335:35.134,334:35.134)1014:35.134)1012:30.9324,((71:76.1545,529:76.1545)1016:8.84186,326:84.9964)1015:16.2041)1011:16.4732)900:1.49186)719:17.735,(((((613:48.5327,614:48.5327,615:48.5327)1021:48.5327,18:97.0653)1020:33.2397,(((509:52.7228,502:52.7228,506:52.7228,504:52.7228,508:52.7228,503:52.7228,505:52.7228)1024:52.7228,644:105.446,6:105.446,(145:52.7228,147:52.7228,146:52.7228,142:52.7228,144:52.7228,141:52.7228,143:52.7228)1025:52.7228,(40:52.7228,39:52.7228)1026:52.7228,(165:97.6117,507:97.6117)1027:7.83397)1023:18.9548,(88:58.6342,87:58.6342,90:58.6342,89:58.6342)1028:65.7662)1022:5.90453)1019:2.57748,(166:56.4912,168:56.4912,170:56.4912,169:56.4912,167:56.4912)1029:76.3913)1018:3.20966,670:136.092)1017:0.808344)718:43.7278,((((59:60.5241,60:60.5241,61:60.5241)1033:60.5241,(66:60.5241,64:60.5241)1034:60.5241,65:121.048,62:121.048)1032:1.10959,((290:29.1878,291:29.1878)1036:29.1878,568:58.3757)1035:63.7822)1031:24.9074,(((263:45.4977,234:45.4977)1039:49.9973,((398:29.9018,399:29.9018)1041:57.455,(417:32.8858,416:32.8858)1042:54.471)1040:8.13828)1038:25.2054,(283:93.8641,362:93.8641)1043:26.8363)1037:26.3648)1030:33.5631)717:7.65496,((((((((((426:35.3463,427:35.3463,428:35.3463)1053:35.3463,(473:35.3463,472:35.3463)1054:35.3463,383:70.6927,361:70.6927)1052:4.933239,198:75.6259)1051:22.2491,325:97.875)1050:25.9891,(175:111.445,238:111.445,3:111.445,424:111.445,(265:55.7225,266:55.7225,264:55.7225,267:55.7225)1056:55.7225,(470:55.7225,467:55.7225,469:55.7225,468:55.7225)1057:55.7225,173:111.445,(359:55.7225,356:55.7225,360:55.7225,357:55.7225,358:55.7225)1058:55.7225,(421:55.7225,422:55.7225)1059:55.7225,133:111.445,(99:55.7225,100:55.7225,101:55.7225,102:55.7225)1060:55.7225,(225:55.7225,224:55.7225)1061:55.7225,(457:55.7225,456:55.7225)1062:55.7225,124:111.445,418:111.445,125:111.445,(713:55.7225,714:55.7225)1063:55.7225,92:111.445,34:111.445,191:111.445,(184:92.1732,(611:81.3961,(((605:48.9296,190:48.9296)1068:6.02719,63:54.9568)1067:4.12479,(179:2.94841,178:2.94841)1069:56.1331)1066:22.3145)1065:10.7771)1064:19.2718)1055:12.4191)1049:1.04654,402:124.911)1048:9.80233,((((599:51.3738,(601:25.5408,600:25.5408)1074:25.833)1073:29.2141,((353:29.2241,352:29.2241,351:29.2241)1076:29.2241,(661:29.2241,660:29.2241,659:29.2241)1077:29.2241)1075:22.1397)1072:25.8718,(204:3.36123,205:3.36123)1078:103.098)1071:6.20871,((275:31.8588,276:31.8588)1080:31.8588,(440:31.8588,441:31.8588)1081:31.8588,(679:31.8588,678:31.8588)1082:31.8588,663:63.7175)1079:48.9509)1070:22.0446)1047:0.152371,((((27:41.5866,29:41.5866,26:41.5866,30:41.5866,31:41.5866,28:41.5866,23:41.5866)1086:41.5866,(32:30.373,(25:14.3141,24:14.3141)1088:16.0588)1087:52.8002)1085:23.7091,206:106.882)1084:1.27196,(((163:36.9394,161:36.9394)1091:36.9394,408:73.8789,162:73.8789)1090:27.0152,458:100.894)1089:7.26022)1083:26.7111)1046:2.66859,20:137.534)1045:20.8081,(((55:61.5908,56:61.5908)1094:61.5908,(58:5.33919,57:5.3392)1095:117.842)1093:21.9487,557:145.13)1092:13.2117)1044:29.9412)716:29.3494,(572:105.401,(312:48.7614,311:48.7614)1097:56.6391)1096:112.232)715;

END;
