# Supplementary figures and images for: Flower colour and size-signals vary with altitude and resulting climate on the tropical-subtropical islands of Taiwan
Source: Front Plant Sci. 2024 Feb 1;15:1304849. doi: 10.3389/fpls.2024.1304849 (PMC10867191; doi:10.3389/fpls.2024.1304849)

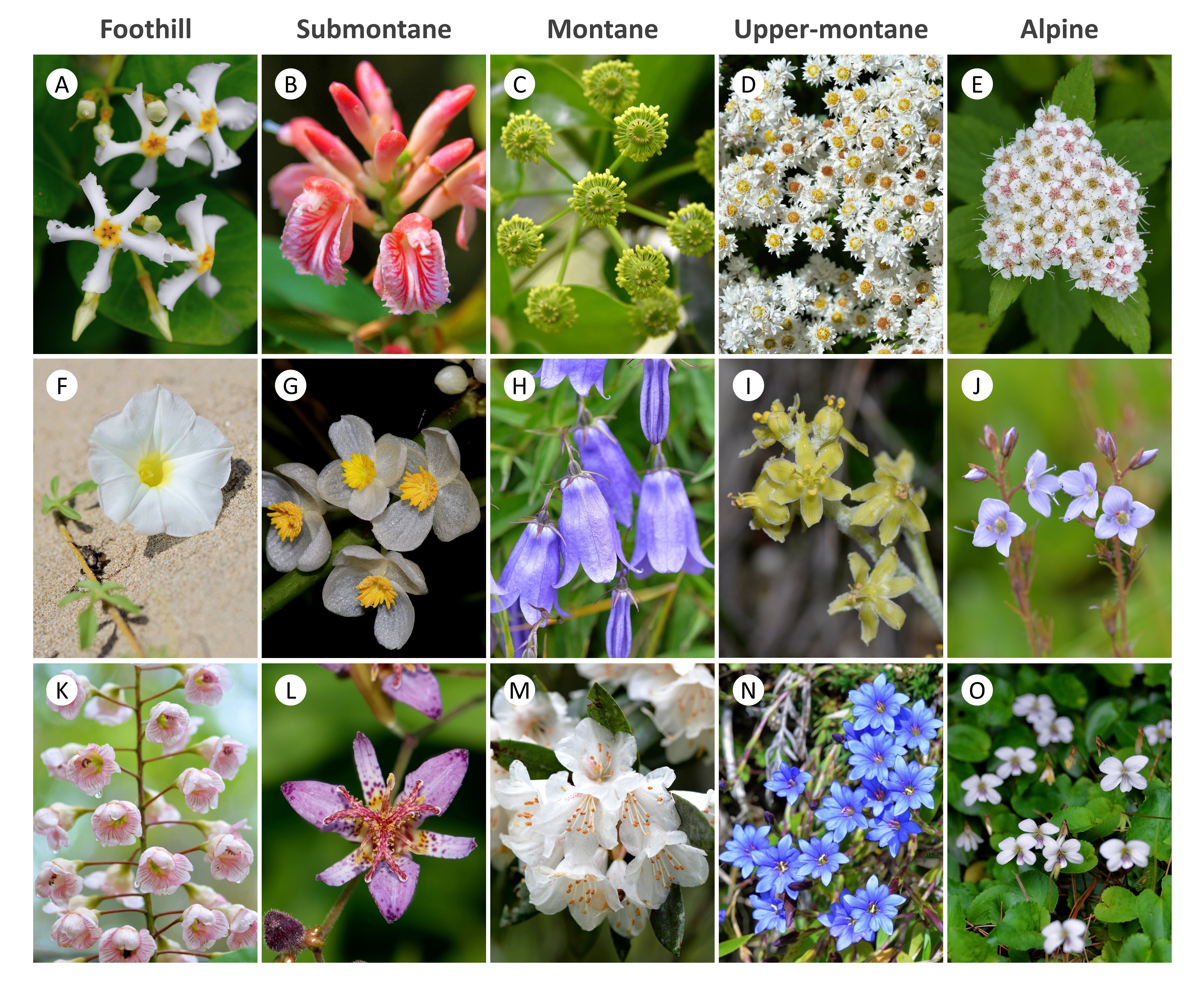

Supplement: Supplementary method S1 — Calculation details for green contrast and colour contrast. [file DataSheet_1.zip › Data_&_figures_RScript/flower examples without scale.jpg]

Foothill

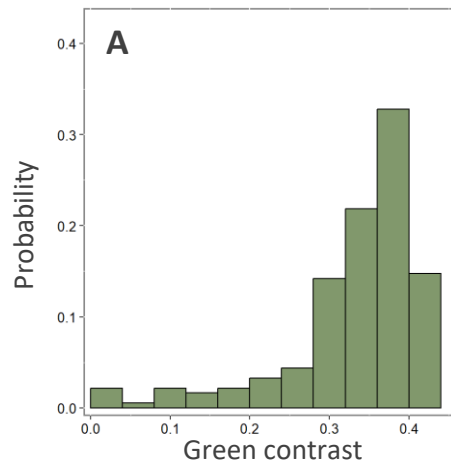

Submontane

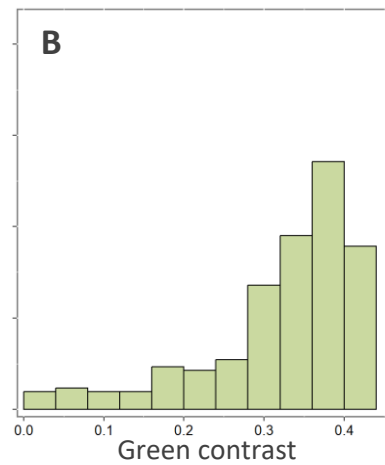

Montane

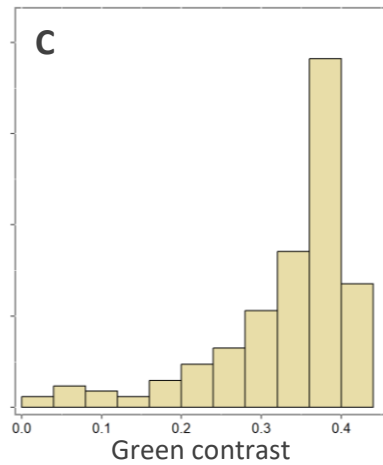

Upper-montane

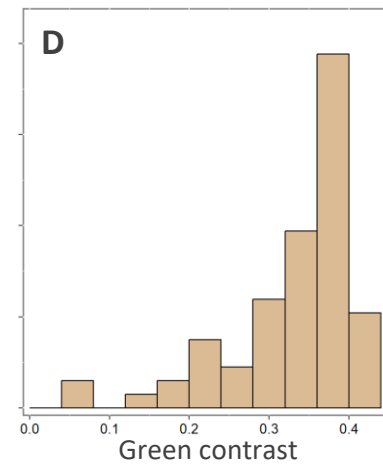

Alpine

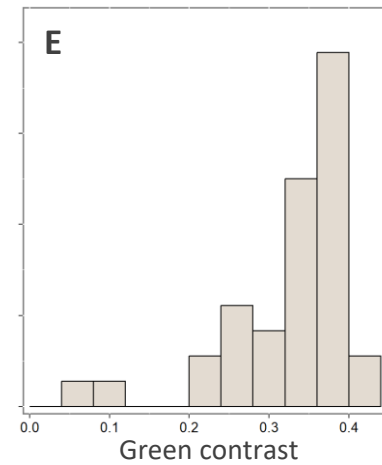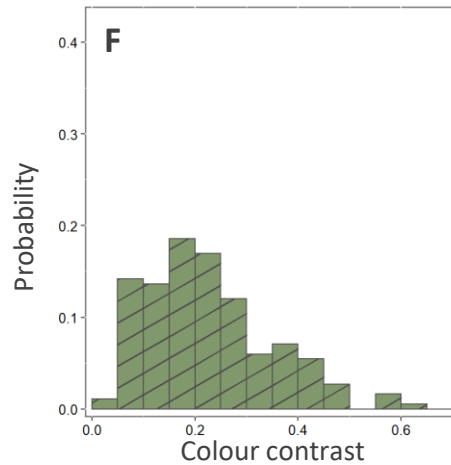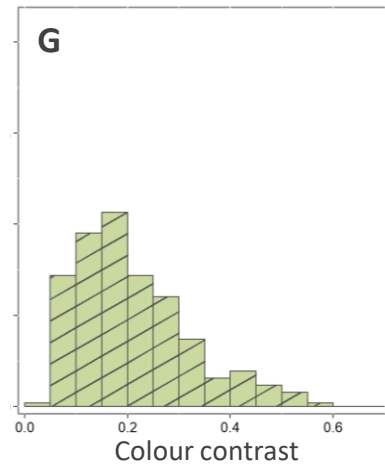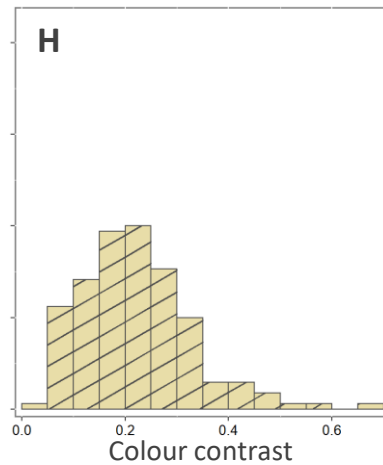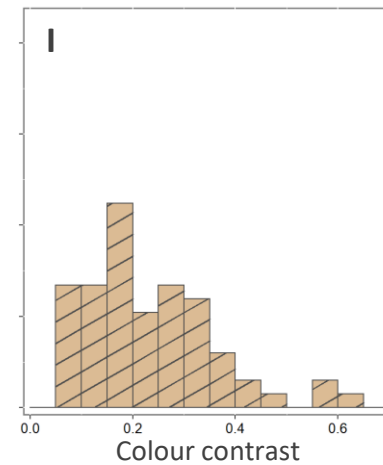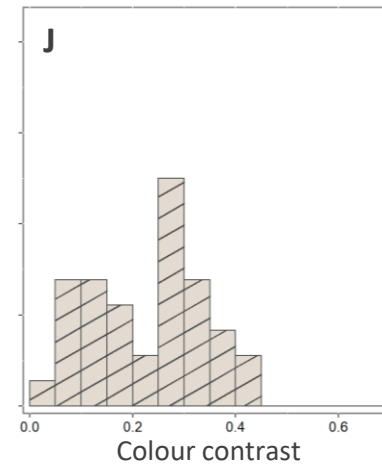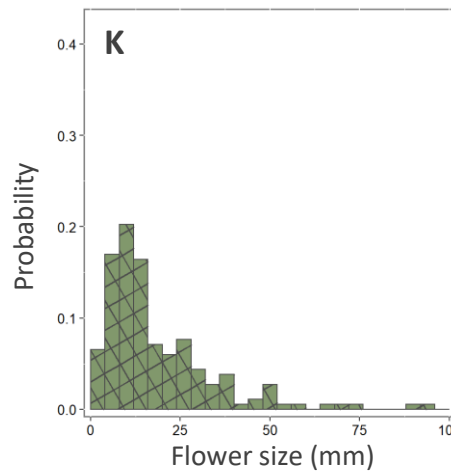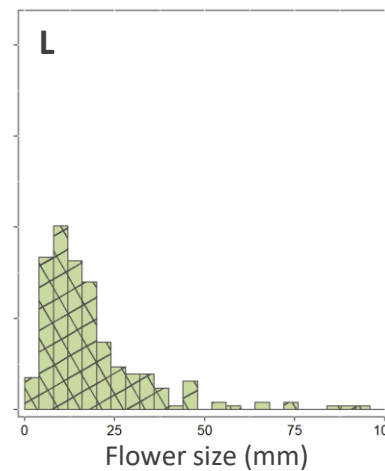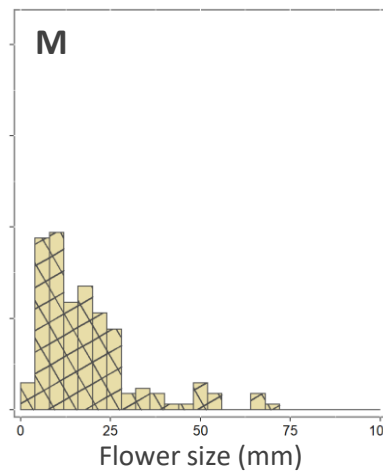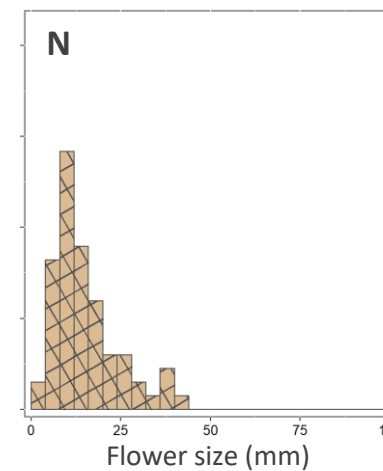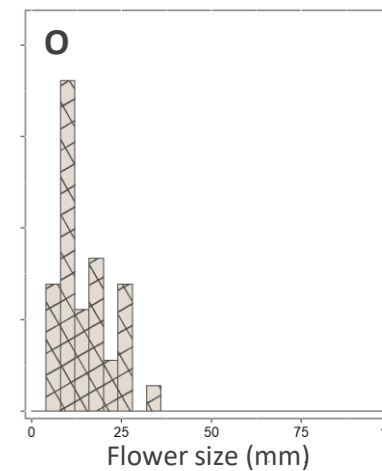

Supplement: Supplementary method S1 — Calculation details for green contrast and colour contrast. [file DataSheet_1.zip › Data_&_figures_RScript/frequency-gc cc size V2.pdf]

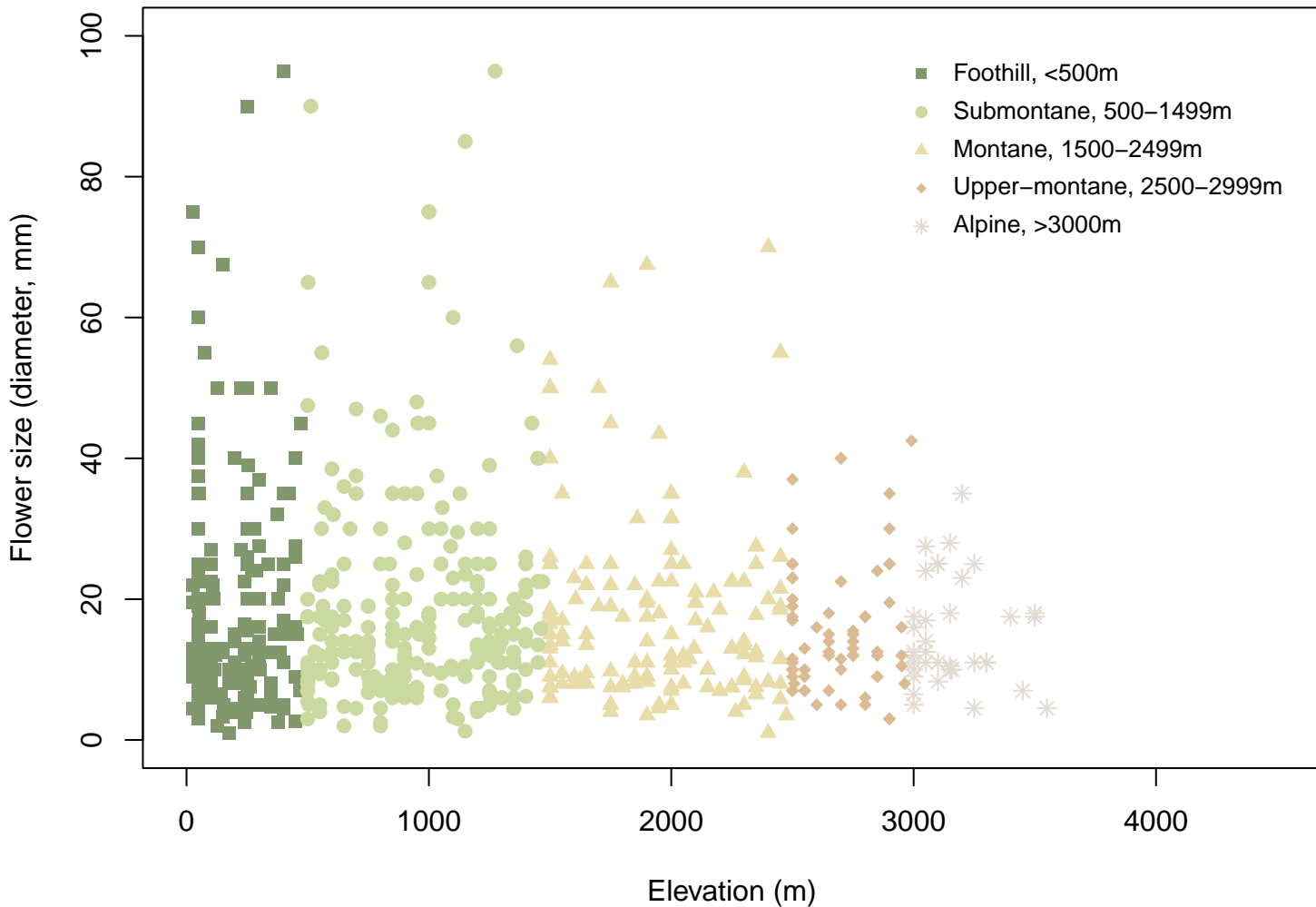

Supplement: Supplementary method S1 — Calculation details for green contrast and colour contrast. [file DataSheet_1.zip › Data_&_figures_RScript/alt vs size.pdf]

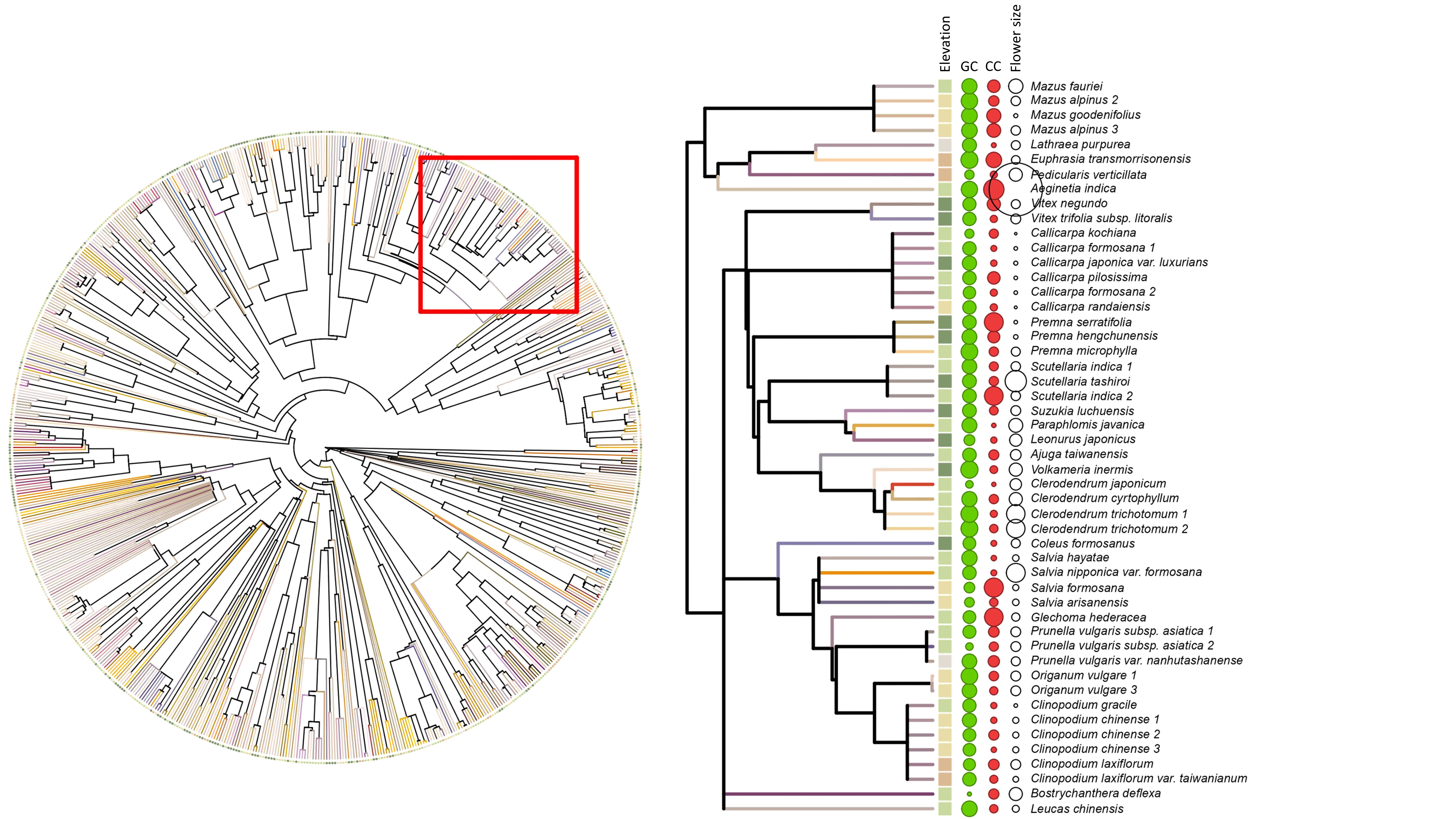

Supplement: Supplementary method S1 — Calculation details for green contrast and colour contrast. [file DataSheet_1.zip › Data_&_figures_RScript/tw50tree and trait 2.jpg]
